# Ferroptosis contributes to ethanol-induced hepatic cell death via labile iron accumulation and GPx4 inactivation

**DOI:** 10.1038/s41420-023-01608-6

**Published:** 2023-08-25

**Authors:** Jiao Luo, Ge Song, Ningning Chen, Mengyue Xie, Xuan Niu, Shuyue Zhou, Yanan Ji, Xiaoxiao Zhu, Wanli Ma, Qianqian Zhang, Dianke Yu

**Affiliations:** School of Public Health, Qingdao University, Qingdao, China

**Keywords:** miRNAs, Cell death

## Abstract

Alcohol abuse is a significant cause of global morbidity and mortality, with alcoholic liver disease (ALD) being a common consequence. The pathogenesis of ALD involves various cellular processes, including oxidative stress, inflammation, and hepatic cell death. Recently, ferroptosis, an iron-dependent form of programmed cell death, has emerged as a potential mechanism in many diseases. However, the specific involvement and regulatory mechanisms of ferroptosis in ALD remain poorly understood. Here we aimed to investigate the presence and mechanism of alcohol-induced ferroptosis and the involvement of miRNAs in regulating ferroptosis sensitivity. Our findings revealed that long-term ethanol feeding induced ferroptosis in male mice, as evidenced by increased expression of ferroptosis-related genes, lipid peroxidation, and labile iron accumulation in the liver. Furthermore, we identified dysregulation of the methionine cycle and transsulfuration pathway, leading to severe glutathione (GSH) exhaustion and indirect deactivation of glutathione peroxidase 4 (GPx4), a critical enzyme in preventing ferroptosis. Additionally, we identified miR-214 as a ferroptosis regulator in ALD, enhancing hepatocyte ferroptosis by transcriptionally activating the expression of ferroptosis-driver genes. Our study provides novel insights into the involvement and regulatory mechanisms of ferroptosis in ALD, highlighting the potential therapeutic implications of targeting ferroptosis and miRNAs in ALD management.

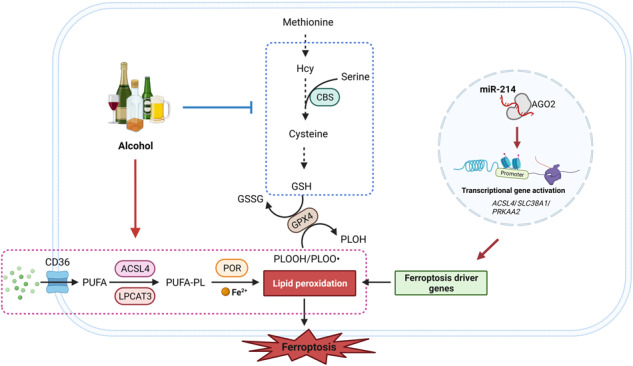

## Introduction

Alcohol is one of the most widely consumed beverages worldwide; however, alcohol abuse remains a major contributor to global morbidity and mortality. The *Global Status Report on Alcohol and Health 2018* indicates that approximately 3.3 million deaths each year are related to alcohol consumption, accounting for 5.9% of all deaths worldwide [1]. Excessive alcohol consumption results in a range of alcoholic liver disease (ALD), from simple alcoholic fatty liver to advanced alcoholic hepatitis, cirrhosis, and eventually hepatocellular carcinoma [2]. Although the pathogenesis of ALD remains insufficiently understood, ethanol metabolism–induced oxidative stress, hepatic cell death, inflammation, etc., have been reported to be involved [3].

Cell death is a natural process required for normal tissue development as well as the removal of damaged and aging cells. However, uncontrolled cell death contributes to the pathogenesis and progression of numerous human diseases, including cancer, neurodegeneration, heart failure, and infectious disease [4]. Hepatocyte death, a prominent pathological feature of ALD, also plays a crucial role in the development of alcoholic liver inflammation and fibrosis [5]. While alcohol exposure can induce various forms of programmed cell death (PCD), such as apoptosis, necroptosis, autophagy, and pyroptosis [6], early studies have reported several ferroptosis-related features, including oxidative stress and iron overload, in patients with alcoholic liver disease [7, 8]. More recently, ferroptosis has been linked to ALD in which Ferrostain-1, a ferroptosis inhibitor, was shown to alleviate alcohol-induced liver injury in female mice fed with Lieber-DeCarli liquid ethanol diet for 10 days [9]. However, another research has shown that ferroptosis features did not appear in male mice fed with an ethanol diet for 10 days [10]. Despite these findings, the concrete evidence of ferroptosis in ALD remains largely unknown.

Ferroptosis is a novel iron-dependent form of PCD, which is widely involved in disease development and toxin-induced tissue injury [11]. Generally, ferroptosis is controlled by glutathione peroxidase 4 (GPx4), the only known enzyme that scavenges lipid hydroperoxides to prevent destructive phospholipid oxidation [12]. However, subsequent research has identified acyl-CoA synthetase long-chain family member 4 (ACSL4) as an essential driver of ferroptosis, which dictates ferroptosis sensitivity, particularly in RSL3-resistant cell lines [13]. In addition to these molecules, multiple proteins have been reported to regulate the process of ferroptosis. For instance, SLC38A1-mediated glutamine uptake promotes ferroptosis and triggers heart injury in ischemia-reperfusion [14], while AMPK-mediated BECN1 phosphorylation promotes ferroptosis by directly blocking system X_c_^–^ activity; inhibition of PRKAA2/AMPKα diminishes ferroptosis [15]. Nevertheless, the regulatory mechanism of ferroptosis in alcohol-induced liver injury is far more than what has been defined at present.

MicroRNAs (miRNAs) are a class of ~22 nt single-strand noncoding RNA that typically inhibit target gene expression [16] or unconventionally modulate transcription [17]. miRNAs play a crucial role in regulating various cellular processes, including proliferation, differentiation, and cell death, and are essential for maintaining tissue development and homeostasis. Dysregulation of miRNA expression has been linked to a variety of human diseases, including cancer and ALD [18, 19]. Some miRNAs have been shown to modulate ferroptosis. For instance, miR-137 negatively regulates ferroptosis by targeting the glutamine transporter SLC1A5 [20], whereas miR-150-5p targets glutamine transporter SLC38A1 [21]. Knockdown of miR-424-5p increases ferroptosis sensitivity of ovarian cancer cells by upregulating ACSL4 expression [22]. Hitherto no miRNAs have been reported to directly regulate ferroptosis in alcoholic liver disease.

This study aims to investigate the involvement and the mechanism underlying alcohol-induced ferroptosis and the role of miRNAs in regulating ferroptosis sensitivity. Long-term ethanol feeding was found to induce labile iron accumulation and enhanced polyunsaturated lipid peroxidation. GPx4 was indirectly deactivated due to GSH exhaustion, which arose as a result of disrupted methionine cycle and dampened transsulfuration pathway. In addition, miR-214 was found to enhance hepatocyte ferroptosis by increasing ferroptosis-driver gene expressions. Our findings provide insight into a potential therapeutic strategy for ALD and highlight the significance of miRNAs in ferroptosis regulation.

## Results

### Ferroptosis occurs in male mice after long-term ethanol feeding

**Fig. 1:**
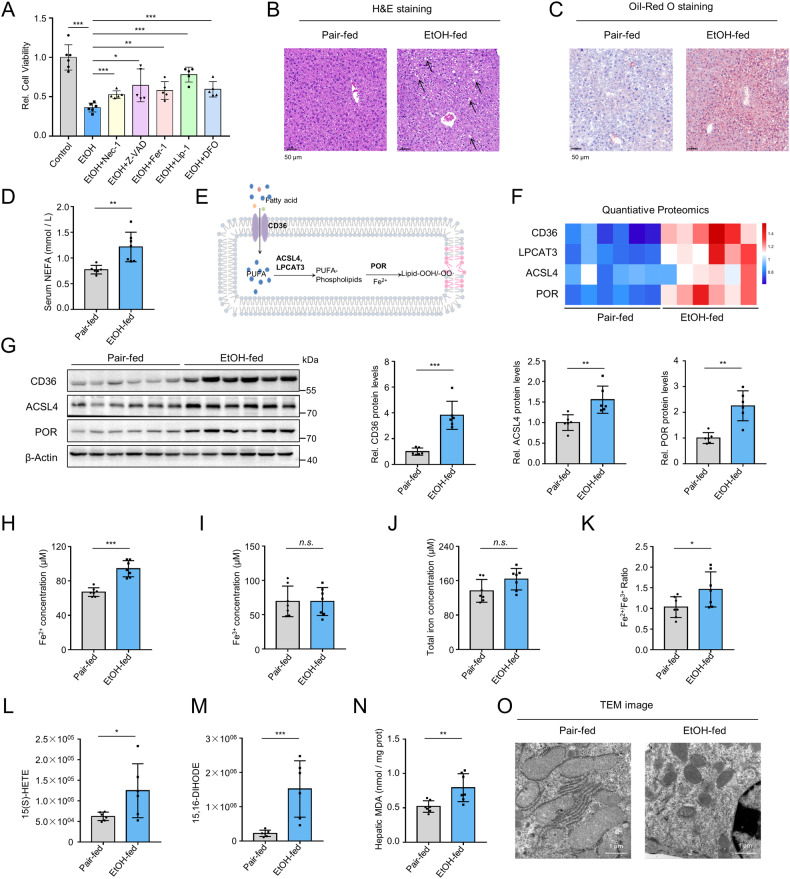
Alcohol induces ferroptosis both in vitro and in vivo. **A** Cell viability analysis of ethanol-exposed AML12 cells pretreated with relative cell death inhibitors. AMl12 cells were pretreated with 25 μM Nec-1, 20 μM Z-VAD-FMK, 1 μM Ferr-1, 1 μM Lip-1, or 10 μM DFO for 3 h, and then cells were exposed to 400 mM ethanol for 24 h in the presence of relative cell death inhibitors. Cell viability was measured using CCK8 assay. **B**, **C** Liver H&E staining (**B**) and Oil-Red O staining (**C**) from mice fed with 8-week ethanol plus one binge (E8w+1B) or pair-fed mice. **D** Serum non-esterified fatty acids (NEFA) levels in E8w+1B mice and pair-fed mice. **E** Figure illustration of the pathway involved in fatty acid uptake and phospholipid hydroperoxide synthesis. **F** Heatmap showing protein levels of CD36, LPCAT3, ACSL4, and POR in the liver of E8w+1B mice and pair-fed mice. The expression profiles were retrieved from our TMT proteomics data. **G** Immunoblotting analysis of the expressions of CD36, LPCAT3, ACSL4, and POR in the liver of E8w+1B mice and pair-fed mice. **H**–**J** Liver concentrations of ferrous irons (**H**), ferric irons (**I**), and total irons (**J**) in E8w+1B mice and pair-fed mice. **K** The ratios of ferrous irons to ferric irons in the liver of E8w+1B mice and pair-fed mice. **L**–**N** The levels of lipid peroxidation products 15(S)-HETE (**L**), 15,16-DiHODE (**M**), and MDA (**N**) in the liver of E8w+1B mice and pair-fed mice. **O** Representative transmission electron microscopy (TEM) images of the liver from E8w+1B mice and pair-fed mice. n.s., not significant; *P < 0.05, **P < 0.01, ***P < 0.001 as indicated.

To investigate whether ethanol is capable of inducing ferroptosis in vitro, AML12 cells were exposed to 400 mM ethanol for 24 h. As shown in Fig. 1A, the loss of cell viability was partially reversed by the necrosis inhibitor Necrostatin-1 (Nec-1) and the apoptosis inhibitor Z-VAD-FMK. However, cell death induced by ethanol was more effectively reversed by the ferroptosis inhibitor Ferrostatin-1 (Fer-1), Liproxstatin-1 (Lip-1), or deferoxamine (DFO), with Lip-1 being the most effective (Fig. 1A). These findings are compatible with the notion that alcohol induces a mixed form of programmed cell death (PCD).

Here, we established a male mouse model fed with an ethanol diet for 8 weeks plus one binge (E8w+1B) (Supplementary Fig. S1A). As illustrated in Fig. 1B, C, the liver from E8w+1B mice appeared bubbled and steatosis, indicating the successful establishment of the ALD model. After long-term ethanol feeding, serum-free fatty acids were found to be significantly increased (Fig. 1D). Core proteins involved in fatty acid uptake and phospholipid hydroperoxide synthesis, including CD36, ACSL4, and NADPH-cytochrome P450 reductase (POR), have been validated as drivers of ferroptosis (Fig. 1E). Both proteomic and immunoblotting data demonstrated that the expression levels of CD36, ACSL4, and POR were markedly increased in E8w+1B mice (Fig. 1F, G). Additionally, the accumulation of polyunsaturated fatty acids (PUFAs), including DHA, DPA, and EPA, was observed in the livers of E8w+1B mice (Supplementary Fig. S1C–F). Ferrous iron, another essential trigger for the occurrence of ferroptosis, was also found to be significantly accumulated in the E8w+1B livers (Fig. 1H). The levels of ferric iron and total iron had no difference between E8w+1B mice and pair-fed mice, however, the ratio of ferrous iron to ferric iron was significantly increased (Fig. 1I–K). Furthermore, the secondary products of lipid peroxidation, 15(*S*)-HETE, 15,16-DiHODE, and MDA, were significantly accumulated in the livers of E8w+1B mice (Fig. 1L–N). Transmission electron microscopy (TEM) images showed the mitochondria were smaller in volume, and the outer membrane was ruptured after long-term ethanol feeding (Fig. 1O). Taken together, these hallmarks suggest that liver ferroptosis is present in male mice after long-term ethanol feeding.

Although the protein levels of CD36, ACSL4, and POR were also upregulated in the liver of mice fed with a 10-day ethanol diet plus one binge (E10d+1B) (Supplementary Fig. S2A–C), no significant difference in lipid peroxidation products was observed between E10d+1B mice and pair-fed mice (Supplementary Fig. S2D, E). Labile iron levels were also unaffected by short-term ethanol feeding (Supplementary Fig. S2F–I). These findings suggested that hepatic ferroptosis cannot be induced by 10-day ethanol feeding in male mice.

### Long-term ethanol feeding indirectly deactivated GPx4 through severe GSH exhaustion

**Fig. 2:**
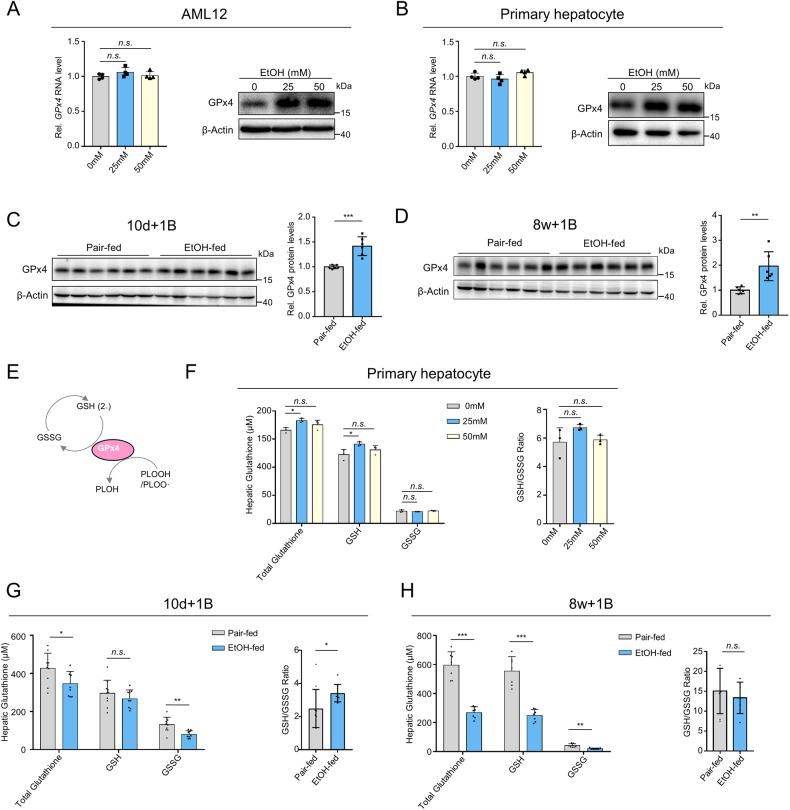
GPx4 upregulation and GSH exhaustion induced by long-term ethanol feeding. **A**, **B** qRT-PCR and immunoblotting analysis of GPX4 expressions in AML12 (**A**) and primary hepatocytes (**B**) exposed to ethanol for 48 h. **C**, **D** Immunoblotting analysis of GPX4 expressions in livers from pair-fed mice and E10d+1B mice (**C**) or E8w+1B mice (**D**). **E** Graphic illustration of GPX4 function in clearance of phospholipid hydroperoxides. **F**–**H** Levels of total glutathione, reductant GSH, and oxidized GSSG in primary hepatocytes exposed to ethanol for 48 h (**F**) or in livers of E10d+1B mice (**G**) or E8w+1B mice (**H**). n.s., not significant; *P < 0.05, **P < 0.01, ***P < 0.001 as indicated.

GPx4 is the only phospholipid hydroperoxide scavenger that protects cells from membrane disruption. We therefore examined how GPx4 expression changed upon ethanol exposure. Strikingly, *GPx4* mRNA levels remained unchanged under ethanol treatment, while GPx4 protein expression was upregulated in both ethanol-exposed AML12 cells and primary hepatic cells (PHC) (Fig. 2A, B). Similar results were observed in the livers of E10d+1B mice (Fig. 2C) and E8w+1B mice (Fig. 2D).

GPx4 detoxifies lipid hydroperoxides using glutathione (GSH) (Fig. 2E). We found that in vitro exposure to the physiological concentration of alcohol did not affect the cellular levels of total glutathione, GSH and GSSG, nor the GSH/GSSG ratios (Fig. 2F). Short-term ethanol feeding did not change the GSH levels but lowered GSSG levels (Fig. 2G). However, long-term ethanol feeding severely dampened hepatic levels of total glutathione, reductant GSH, and GSSG (Fig. 2H). Altogether, our findings indicate that GPx4 activity is indirectly deactivated through the unavailability of GSH after long-term ethanol treatment.

### Chronic ethanol treatment disrupts the methionine cycle and dampens the transsulfuration pathway

**Fig. 3:**
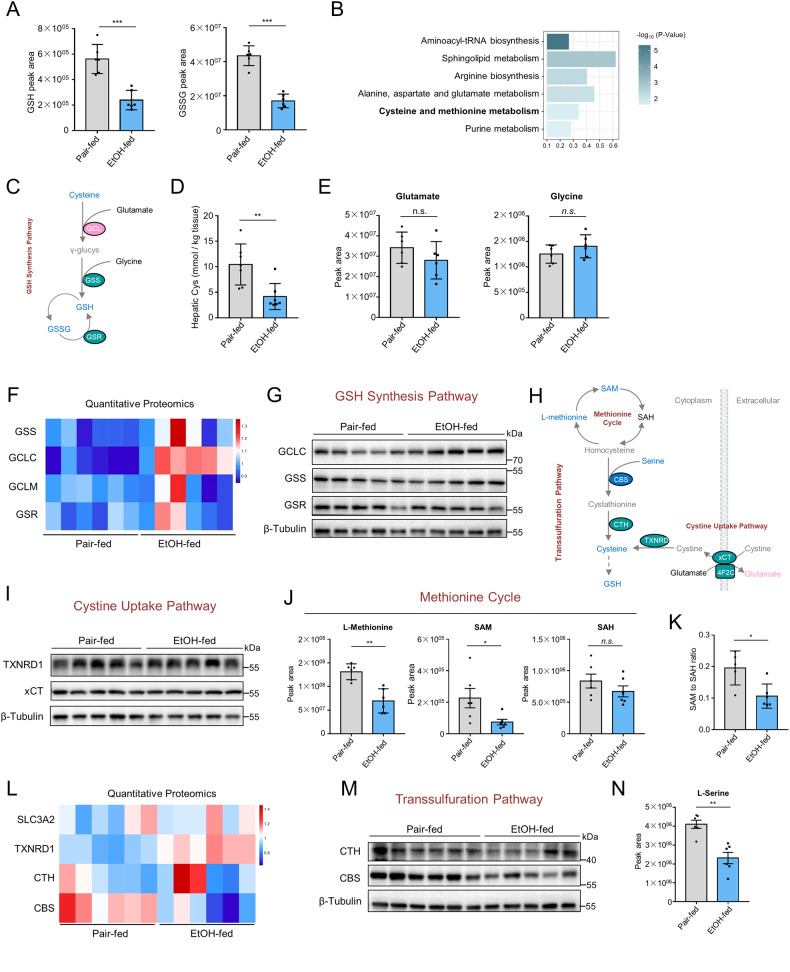
Disrupted methionine cycle and transsulfuration pathway contribute to GSH exhaustion. **A** Hepatic GSH and GSSG levels in E8w+1B mice. The peak area was retrieved from the untargeted metabolomics data. **B** Pathway enrichment analysis of differential metabolites between E8w+1B mice and pair-fed mice. **C** Graphic illustration of the GSH synthesis pathway. **D** Hepatic cysteine levels detected using a Cysteine Colorimetric Assay Kit. **E** Hepatic glutamate and glycine levels in E8w+1B mice. The peak area was retrieved from the untargeted metabolomics data. **F** The expression heatmap of GSS, GCLC, GCLM, and GSR in E8w+1B mice and pair-fed mice. The expression profiles were retrieved from our TMT proteomics data. **G** Immunoblotting analysis of GSS, GCLC, GCLM, and GSR expressions in E8w+1B mice and pair-fed mice. **H** Graphic illustration of cystine uptake pathway, methionine cycle, and transsulfurration pathway. **I** Immunoblotting analysis of xCT and TXNRD1 expressions in E8w+1B mice and pair-fed mice. **J** Hepatic methionine, SAM, and SAH levels in E8w+1B mice. The peak area was retrieved from the untargeted metabolomics data. **K** The ratio of hepatic SAM to SAH. **L** Immunoblotting analysis of CTH and CBS expressions in E8w+1B mice and pair-fed mice. **M** Hepatic serine levels in E8w+1B mice. The peak area was retrieved from the untargeted metabolomics data. *n.s*., not significant; *P < 0.05, **P < 0.01, ***P < 0.001 as indicated. **N** Hepatic serine levels in E8w+1B mice. The peak area was retrieved from the untargeted metabolomics data.

To further elucidate the mechanism underlying ethanol-induced GSH exhaustion, untargeted metabolomics was performed using livers from mice fed a long-term ethanol diet plus one binge (E8w+1B) and pair-fed mice. The decrease of reductant GSH and oxidized GSSG were validated by the metabolomics data (Fig. 3A). Pathway enrichment analysis showed that cysteine and methionine metabolism was enriched after long-term ethanol treatment (Fig. 3B). The GSH synthetic pathway is illustrated in Fig. 3C, where we found that cysteine, the rate-limiting substrate for GSH synthesis, was significantly decreased in E8w+1B mice (Fig. 3D) while other adjuvants like glutamate and glycine were not changed by ethanol (Fig. 3E). Additionally, glutamate-cysteine ligase (GCL) was upregulated, whereas other enzymes in the GSH synthesis pathway were not affected by ethanol (Fig. 3F, G). Our data suggest that the decreased cysteine levels result in GSH unavailability after long-term ethanol consumption.

To investigate the reason for the declined cysteine levels in E8w+1B mice, we analyzed whether ethanol disrupted exogenous cystine uptake or endogenous cysteine biotransformation (Fig. 3H). We found that the expressions of cystine transporter xCT and TXNRD1 proteins were unchanged by ethanol treatment (Fig. 3I). However, chronic ethanol consumption decreased the hepatic levels of methionine and S-adenosylmethionine (SAM), while the level of S-adenosylhomocysteine (SAH) remained unaffected (Fig. 3J), leading to a significantly decreased SAM-to-SAH ratio (Fig. 3K). Furthermore, immunoblotting data revealed a decreased expression of CBS (Fig. 3L), while metabolomic data showed a significant decrease in hepatic serine levels (Fig. 3M). These data suggest that ethanol decreases cysteine through a disrupted methionine cycle and transsulfuration pathway, ultimately leading to GSH exhaustion.

### GPx4 inactivation alone is insufficient to induce ferroptosis in hepatocytes

**Fig. 4:**
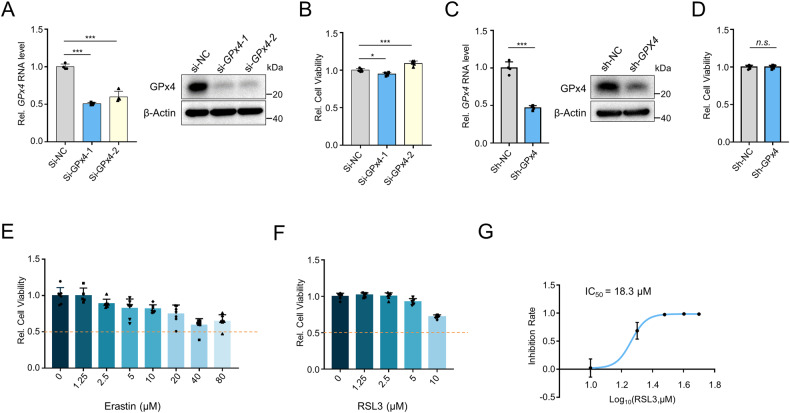
GPx4 inactivation alone is not sufficient to induce ferroptosis in hepatocytes. **A** qRT-PCR and immunoblotting analysis of GPX4 expression in AML12 cells transfected with GPx4 siRNAs (50 nM) for 48 h. **B** Cell viability of AML12 cells transfected with GPx4 siRNAs (50 nM) for 48 h. **C** qRT-PCR and immunoblotting analysis of GPx4 expression in AML12 cells infected with pLKO.1-shGPx4 lentivirus or control lentivirus. After puromycin selection, infected AML12 cells were used to measure cell viability by CCK8 assay. **D** Cell viability analysis of shGPx4 AML12 cells and control cells. **E**, **F** Cell viability of AML12 cells treated with Erastin (**E**) or RSL3 (**F**) for 24 h. **G** IC_50_ value of RSL3 on AML12 cell viability.

GPx4 inactivation is known to have detrimental and even lethal effects on many cells and tissues [23]. To investigate the effect of GPx4 inactivation on hepatic cell viability, we knocked down GPx4 using siRNAs or shRNA lentivirus in AML12 cells, which are derived from normal mouse hepatocytes (Fig. 4A, C). Surprisingly, we observed no significant difference in cell viability between GPx4-knockdown cells and control cells (Fig. 4B, D). In addition, we used GPx4 inhibitors RSL3 and Erastin to bypass GPx4 knockdown for ferroptosis induction. RSL3 directly inactivates GPx4 by covalently binding GPx4 [12], while Erastin indirectly deactivates GPx4 by inhibiting cystine uptake [24]. Generally, concentrations of 10 μM Erastin and 0.1 μM RSL3 are lethal to ferroptosis-sensitive cells such as HT-1080 and Pfa1 cells. However, concentrations up to 80 μM Erastin or 10 μM RSL3 induced less than 50% cell death (Fig. 4E, F). The half-maximal inhibitory concentration (IC_50_) of RSL3 in AML12 cells is 18.3 μM (Fig. 4G). These findings suggest that AML12 cells exhibit resistance to ferroptosis. Similarly, two other hepatocarcinoma cell lines, HepG2 and Hep3B (Supplementary Fig. S4), also demonstrated insensitivity to GPx4 inactivation. Overall, these results indicate that GPx4 inactivation alone is insufficient to induce hepatic ferroptosis.

### miR-214 sensitizes hepatocytes to ferroptosis

**Fig. 5:**
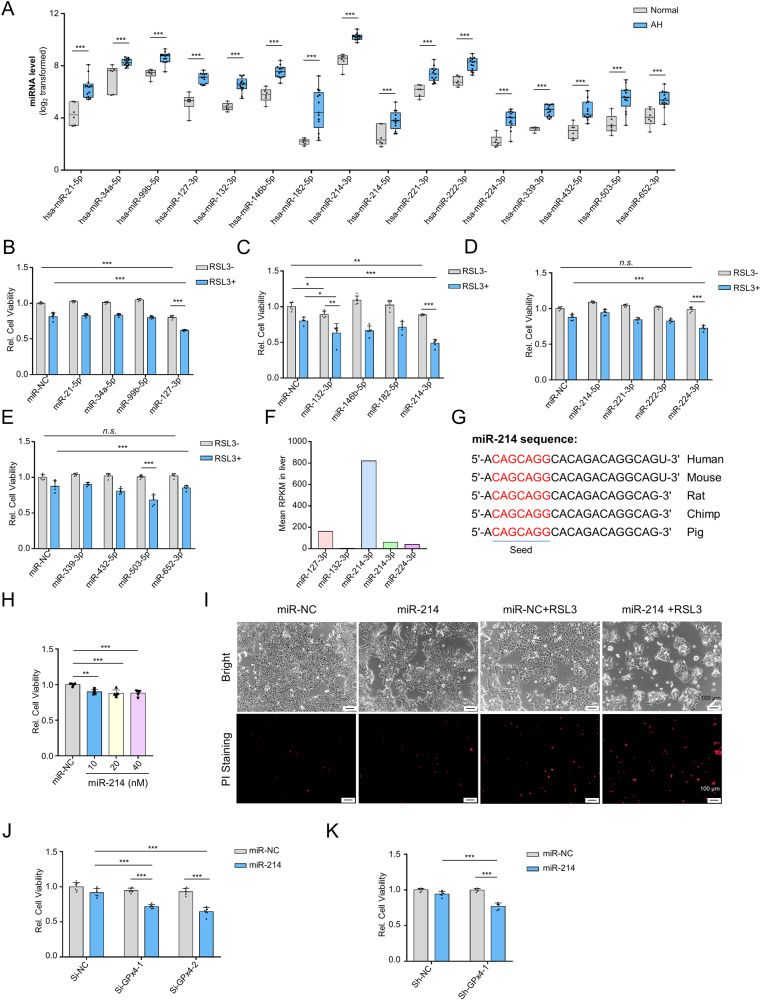
miR-214 sensitizes hepatocytes to ferroptosis. **A** The log_2_-transformed expression values of 16 conserved miRNAs (among the top 25 upregulated miRNAs) in the liver tissues of healthy individuals and alcoholic hepatitis patients. The expression profiles were retrieved from the GSE59492 dataset. **B**–**E** An unbiased screen of miRNAs regulating the sensitivity of AML12 cells to ferroptosis. Cells were transfected with 20 nM miRNA mimics for 24 h and then treated with 10 μM RSL3 for another 24 h. **F** Mean expression levels of miR-127-3p, miR-132-3p, miR-214-3p, miR-224-3p, and miR-503-5p in human liver tissues. The expression profiles were retrieved from the miRmine database. **G** Sequence alignment analysis of miR-214 in humans and other placental mammals. **H** CCK8 analysis of cell viability in AML12 cells transfected with miR-214 mimics for 48 h. **I** The morphology image and PI staining image of AML12 cells transfected with 20 nM miR-214 mimics for 24 h and then treated with 10 μM RSL3 for another 24 h. **J** The effect of miR-214 on the cell viability of *GPX4*-knockdown AML12 cells. AML12 cells were co-transfected with 20 nM miR-214 mimics and/or 50 nM *GPX4* siRNAs for 48 h. **K** The cell viability of shGPX4 AML12 cells transfected with 20 nM miR-214 mimics for 48 h. *n.s*., not significant; *P < 0.05, **P < 0.01, ***P < 0.001 as indicated.

To investigate the biological factors responsible for regulating hepatocyte sensitivity to ferroptosis, we focused on the top 25 upregulated miRNAs in the livers of alcoholic hepatitis (AH) patients from the Gene Expression Omnibus database (accession number GSE59492). A subset of 16 was found to be conserved between humans and mice (Fig. 5A and Supplementary Table S1). We then conducted an unbiased screen of miRNAs to determine their effect on RSL3-induced ferroptosis and identified five miRNAs—miR-127-3p, miR-132-3p, miR-214-3p, miR-224-3p, and miR-503-5p—that could enhance the sensitivity of hepatocytes to ferroptosis (Fig. 5B–E). Of these 5 miRNAs, miR-214-3p (referred to as miR-214) exhibited the highest expression levels in human liver tissues (Fig. 5F) and was conserved across various viviparous animals (Fig. 5G), making it a prime candidate for further investigation.

Although miR-214 alone had little effect on the cell viability of AML12 cells (Fig. 5H), it was access to induce loss of cell viability and plasma membrane disruption (indicated by positive PI staining) in the presence of GPx4 inactivation (Fig. 5I). Additionally, miR-214 significantly reduced the cell viability of GPx4-knockdown AML12 cells (Fig. 5J, K). Our findings indicate that miR-214 increases the susceptibility of hepatocytes to ferroptosis.

### miR-214 promotes the expressions of ferroptosis-driver genes

**Fig. 6:**
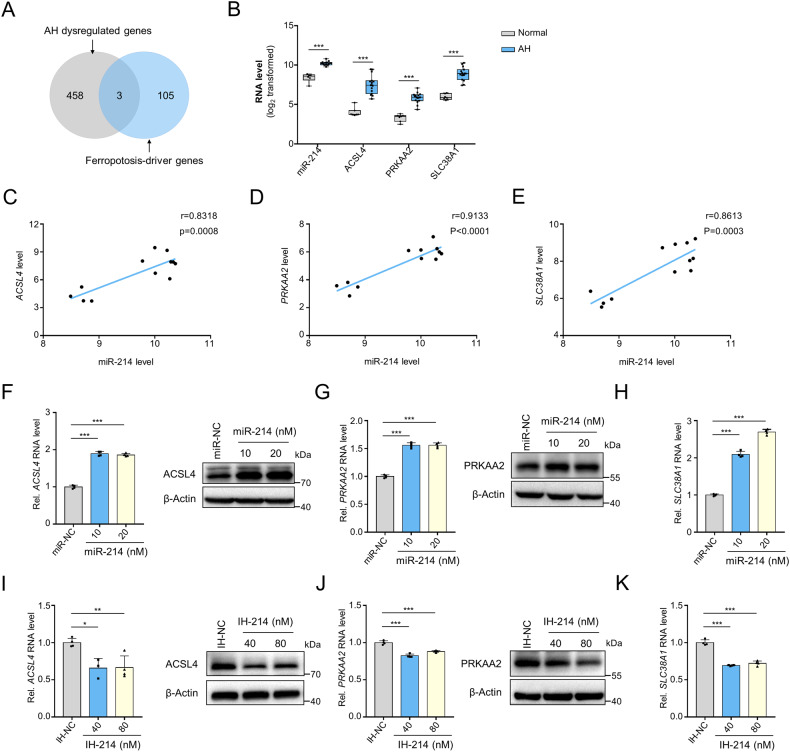
miR-214 upregulates the expressions of ferroptosis-driver genes. **A** Screening of significantly dysregulated ferroptosis-driver genes in alcoholic hepatitis. **B** The log_2_-transformed expression values of *ACSL4*, *PRKAA2*, and *SLC38A1* in the liver tissues of healthy individuals and alcoholic hepatitis patients. The expression profiles were retrieved from the GSE28619 dataset. **C**–**E** Person correlation analysis between the expressions of miR-214 and *ACSL4* (**C**), *SLC38A1* (**D**), and *PRKAA2* (**E**) in the livers of alcoholic hepatitis and healthy individuals. **F**, **G** *q*RT-PCR and immunoblotting analysis of the expression of ACSL4 (**F**) and PRKAA2 (**G**) in primary hepatocytes transfected with miR-214 mimics for 48 h. **H** *q*RT-PCR analysis of *SLC38A1* mRNA expression in primary hepatocytes transfected with miR-214 mimics for 48 h. **I**, **J** *q*RT-PCR and immunoblotting analysis of the expression of ACSL4 (**I**) and PRKAA2 (**J**) in primary hepatocytes transfected with miR-214 inhibitors for 48 h. **K** *q*RT-PCR analysis of *SLC38A1* mRNA expression in primary hepatocytes transfected with miR-214 inhibitors for 48 h. *P < 0.05, **P < 0.01, and ***P < 0.001 as indicated.

To investigate the mechanism underlying increased ferroptosis sensitivity induced by miR-214, we downloaded a gene list of validated ferroptosis-driver genes from the FerrDb database and identified three significantly upregulated ferroptosis-driver genes, *ACSL4*, *SLC38A1*, and *PRKAA2*, in the livers of AH patients (Fig. 6A, B). Pearson correlation analysis demonstrated a highly positive correlation between miR-214 expression and the expression of *ACSL4*, *SLC38A1*, and *PRKAA2* in liver tissues of AH patients and healthy individuals (Fig. 6C–E).

As miRNA generally downregulates its target genes with negative correlations, we hypothesized that miR-214 might unconventionally promote the expression of *ACSL4*, *SLC38A1*, and *PRKAA2*. We then performed qRT-PCR and immunoblotting experiments to validate this hypothesis. The results showed that miR-214 mimics significantly increased the mRNA and protein levels of ACSL4 and PRKAA2 in primary hepatocytes (Fig. 6F–H). Unfortunately, effective antibodies for SLC38A1 were not available, so we did not present the immunoblotting results for this protein here. Additionally, treatment with miR-214 inhibitors inhibited the expression levels of *ACSL4*, *SLC38A1*, and *PRKAA2* mRNAs, as well as reduced the protein levels of ACSL4 and PRKAA2 (Fig. 6I–K). Taken together, our findings suggest that miR-214 promotes the expression of ferroptosis genes ACSL4, SLC38A1, and PRKAA2 in hepatocytes.

### miR-214 promotes transcriptional activation of ferroptosis-driver genes

**Fig. 7:**
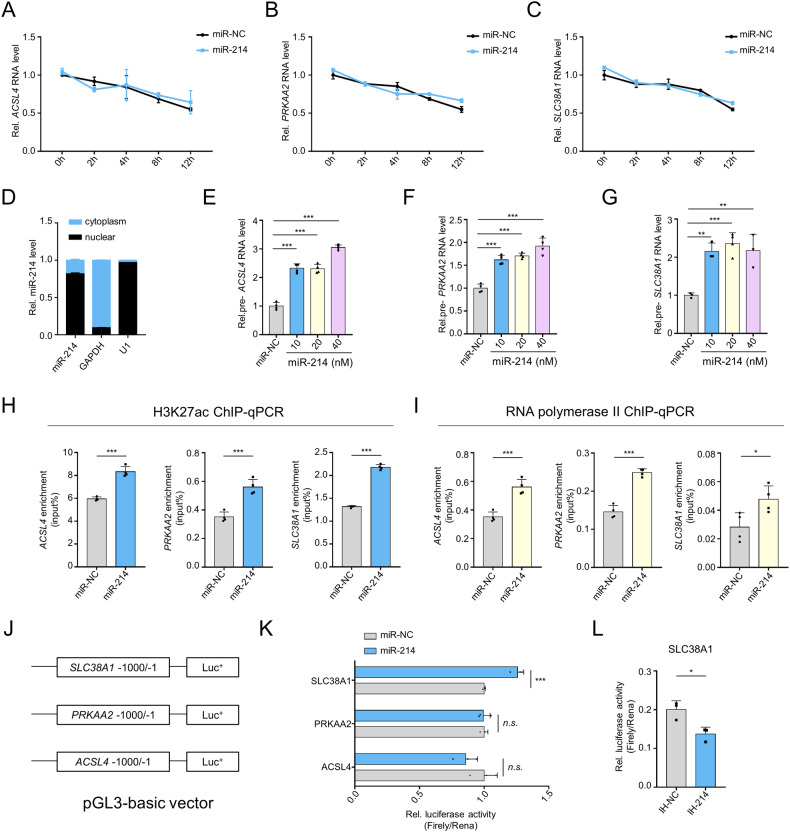
miR-214 transcriptionally activates ferroptosis-driver genes. **A**–**C** qRT-PCR analysis of mRNA stability of *ACSL4* (**A**), *PRKAA2* (**B**), and *SLC38A1* (**C**) in miR-214-transfected primary hepatocytes. **D** Cell fraction analysis of miR-214 in AML12 cells. *U1* and *GAPDH* were used as nuclear and cytoplasmic markers, respectively. **E**–**G** *q*RT-PCR analysis of the primary RNA levels of *ACSL4* (**E**), *PRKAA2* (**F**), and *SLC38A1* (**G**) in primary hepatocytes transfected with miR-214 mimics for 48 h. **H**, **I** ChIP-qPCR analysis of the enrichment of histone H3K27ac (**H**) and RNA polymerase II (**I**) at the promoter region of *ACSL4*, *SLC38A1*, and *PRKAA2* gene. Primary hepatocytes were transfected with miR-214 mimics (20 nM) for 48 h, and then cells were applied to the ChIP assay. **J** Schematic illustration of the luciferase reporter construction that detects *ACSL4*/*SLC38A1*/*PRKAA2*-drived promoter activities in response to miR-214 treatment. **K** The promoter activities measured by the luciferase reporter assay in HEK-293FT cells in response to miR-214 mimics (20 nM). **L** Luciferase reporter assay of *SLC38A1*-drived promoter activity after miR-214 inhibitor (40 nM) treatment. n.s., not significant; *P < 0.05, **P < 0.01, ***P < 0.001 as indicated.

To investigate how miR-214 upregulates the expression of ACSL4, SLC38A1, and PRKAA2, we first analyzed their RNA stability. Our qRT-PCR results showed that miR-214 did not affect the mRNA degradation of *ACSL4*, *SLC38A1*, and *PRKAA2* (Fig. 7A–C) and also failed to block the degradation of their primary transcripts (Supplementary Fig. S5A–C), indicating that miR-214 has no effect on the stability of mRNA and primary RNA transcripts. Cell fraction experiments revealed that miR-214 is mainly located in the nucleus (Fig. 7D), suggesting its potential function in the nucleus. Notably, the primary transcript levels of *ACSL4*, *SLC38A1*, and *PRKAA2* were significantly upregulated by miR-214 mimics (Fig. 7E–G). H3K27ac modification is closely associated with transcriptional gene activation, and ChIP-qPCR data showed that miR-214 increased the enrichment of H3K27ac at the proximal promoter regions of *ACSL4*, *SLC38A1* and *PRKAA2* (Fig. 7H), suggesting increased accessibility of promoter chromatin. Furthermore, RNA polymerase II (RNAP II) was found to be enriched at the promoter regions of target genes by miR-214 (Fig. 7G–I), suggesting that miR-214 could promote the assembly of the transcriptional complex in the promoter regions of target genes, leading to the transcriptional activation of ferroptosis-driver genes.

To further determine whether miR-214 directly targets the promoter regions, a 1 kb promoter sequence upstream of the transcriptional start site of *ACSL4*, *SLC38A1*, and *PRKAA2* was inserted into a pGL3-basic vector, respectively (Fig. 7J). Our results showed that miR-214 significantly increased the luciferase activity driven by the *SLC38A1* promoter (Fig. 7K), while endogenous miR-214 inhibition inhibited *SLC38A1* promoter-stimulated luciferase activity (Fig. 7L). However, miR-214 had no effect on the luciferase activity driven by *PRKAA2* and *ACSL4* promoter, indicating that miR-214 does not directly target the proximal promoter regions of *PRKAA2* and *ACSL4*. The special target sites require further investigation in the future.

### miR-214 selectively loads into nuclear AGO2 to upregulate ferroptosis-driver genes

**Fig. 8:**
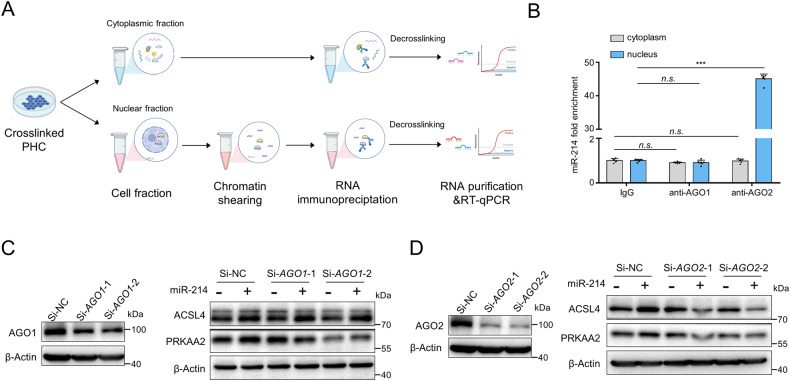
AGO2 participates in the positive regulation of miR-214. **A** Schematic illustration of RIP analysis of the interaction between miR-214 and AGO1 or AGO2 proteins in the cytoplasm and nucleus of mouse primary hepatocytes. **B** RIP analysis of miR-214 enrichment in the cytoplasm and nucleus using AGO1 and AGO2 antibody. Immunoblotting analysis of the expressions of ACSL4 and PRKAA2 protein in primary hepatocytes co-transfected with miR-214 mimics (20 nM) and/or AGO1 siRNAs (**C**) or AGO2 siRNAs (**D**). Data are expressed as means ± SD from at least three independent experiments. ns not significant; *P < 0.05, **P < 0.01, and ***P < 0.001.

AGO1 and AGO2 proteins are typically involved in nuclear miRNA-mediated gene silencing or activation. Here we fractioned the nuclear and cytoplasmic components of primary hepatocytes and performed RNA immunoprecipitation using specific antibodies against AGO1 or AGO2 (Fig. 8A). Results showed that endogenous miR-214 loaded specifically into AGO2 in the nucleus (with about 45-fold enrichment), but not into AGO1 or AGO2 in the cytoplasm (Fig. 8B). To evaluate the role of AGO1/2 in the miR-214-induced expression of ferroptosis-driver genes, siRNAs targeting *AGO1* and *AGO2* were used to knock down their expressions. We found that miR-214 could still upregulate ACSL4 and PRKAA2 expression in AGO1-knockdown hepatocytes (Fig. 8C), whereas AGO2-knockdown abrogated this effect (Fig. 8D). Our findings indicate that miR-214 was loaded into AGO2 proteins in the nucleus to upregulate ferroptosis-driver genes.

## Discussion

Our study aimed to investigate the association between ferroptosis and alcoholic liver disease. We have uncovered several significant findings, including (1) Ferroptosis is involved in ethanol-induced cell death, wherein labile iron and lipid peroxidation products accumulate in the liver; (2) Chronic ethanol consumption indirectly inactivates GPx4 through severe GSH depletion; (3) Chronic ethanol exposure disrupts the methionine cycle and dampens the transsulfuration pathway, resulting in decreased cysteine levels and ultimately contributing to GSH exhaustion; (4) Inactivation of GPx4 alone is insufficient to induce ferroptosis in hepatocytes; (5) miR-214 helps sensitize hepatocytes to ferroptosis by upregulating the transcription of key ferroptosis-driver genes, including *ACSL4*, *SLC38A1*, and *RPKAA2*. These observations provide critical insights into the underlying mechanisms of alcoholic liver disease, highlighting the significance of ferroptosis in disease development and identifying potential therapeutic strategies for prevention and management.

Ferroptosis is a type of necrotic cell death that can be suppressed by lipid peroxidation inhibitors and iron chelators [25]. We observed that AML12 cells exhibited high resistance to ethanol exposure (data not shown), with significant decreases in cell viability only occurring at high concentrations of ethanol (400 mM in this study). We found that the lipid peroxidation inhibitors Fer-1 and Lip-1, as well as the iron chelator DFO, effectively reversed ethanol-induced cell death, indicating the involvement of ferroptosis in alcohol-induced liver injury. Furthermore, Z-VAD-FMK, an apoptosis inhibitor, and Nec-1, a necrosis inhibitor, also improved cell viability under ethanol exposure. These results suggest that alcohol and its metabolite acetaldehyde cause various forms of hepatic cell death [26]. Moreover, we found that the ferroptosis inhibitor Lip-1 was more effective in ameliorating cell death induced by heavy ethanol, making it a promising therapeutic candidate for ethanol-induced liver injury.

To investigate whether ferroptosis is involved in alcohol-induced liver injury in male mice, we extended the ethanol feeding time to 8 weeks and administered a single dose of ethanol gavage on the last day. The hallmarks of ferroptosis were significantly present in the livers of 8w+1B male mice, including increased ferrous irons, unrestrained lipid peroxidation, and shrunken mitochondrial. However, these features were absent in the livers of 10d+1B male mice. These findings underscore the importance of alcohol consumption duration in the occurrence of ferroptosis in male mice.

Overwhelming lipid peroxidation is a characteristic feature of ferroptosis. However, the proteins responsible for phospholipid hydroperoxides (PLOOHs) synthesis, including CD36, ACSL4, and POR, were increased in the livers of both 10d+1B mice and 8w+1B mice. This finding further highlights the crucial role of ferrous iron overload in alcohol-induced ferroptosis, as H_2_O_2_ generated by POR initiates a Fenton reaction with labile iron to produce hydroxyl radicals, leading to lipid peroxidation [27]. Chronic ethanol feeding resulted in ferrous iron accumulation in the liver, suggesting that iron-chelating agents such as FDA-approved Deferoxamine (DFO), Deferiprone (DFP), and Deferasirox (DFX) can be used to ameliorate alcohol-induced liver injury by inhibiting ferroptotic cell death. Further investigations will be conducted in future studies.

GPx4 is widely recognized as the gatekeeper for ferroptosis, which utilizes GSH to selectively detoxify lipid peroxides [12]. Surprisingly, our study found that ethanol upregulated GPx4 protein levels both in vitro and in vivo. However, GPx4 activity was controlled by GSH, which was severely depleted by chronic ethanol feeding. This finding suggests that GPx4 inactivation may synergize with ferrous iron overload to facilitate ferroptosis in alcoholic liver disease. Furthermore, ethanol exposure did not affect GPx4 mRNA expression, indicating the existence of post-transcriptional regulation for GPx4 expression under ethanol treatment.

Our untargeted metabolomic data analysis revealed disturbances in cysteine and methionine metabolism following long-term ethanol treatment. We further found that the disrupted methionine cycle and dampened transsulfuration pathway resulted in decreased cysteine levels in the liver, which ultimately contributes to GSH unavailability in chronic alcoholic liver injury. Notably, the expression levels of proteins involved in cysteine-to-GSH synthesis were either unchanged or upregulated in the livers of E8w+1B mice. Supplementation with cysteine or its analogs, such as N-acetylcysteine, may help to maintain hepatic GSH levels after prolonged ethanol consumption and hold therapeutic potential. Further investigations are required to explore the possible benefits of such interventions.

Ferroptosis is tightly regulated by GPx4 [12, 28]. Inactivation or downregulation of GPx4 is known to induce increased lipid peroxidation and ferroptotic cell death. However, our study found that genetic or pharmacologic GPx4 inactivation was not effective in inducing ferroptosis in hepatocytes, indicating their inherent resistance to this process. This observation is consistent with previous studies showing that the sensitivity to ferroptosis varies across different cell types [13, 29]. It also suggests the presence of additional factors that may modulate hepatocyte susceptibility to ferroptosis in parallel with GPx4.

miRNAs play a crucial role in regulating various biological processes, including cell proliferation, differentiation, and death, with some miRNAs reported to modulate ferroptosis sensitivity. For instance, inhibition of miR-137 and miR-9 enhanced RSL3-induced ferroptosis in melanoma by targeting SLC5A1 and GOT1, respectively, in the glutamine metabolism pathway [20, 30]. Similarly, the upregulation of miR-424-5p reduced the sensitivity of HO8910 and SKOV3 ovarian cancer cells to RSL3-induced ferroptosis by inhibiting ACSL4 [22]. In our study, by screening a miRNA library, we found that miR-214, which was significantly upregulated in the livers of alcoholic hepatitis patients, promoted ferroptosis in AML12 cells in the presence of GPx4 inactivation. These findings suggest that inhibition of miR-214 may be a viable therapeutic option for preventing or treating alcohol-induced liver damage.

miR-214 was initially identified as a tumor promoter through *PTEN* targeting in human ovarian cancer [31]. Subsequent studies revealed its role in inhibiting bone formation by regulating ATF4 [32] and promoting hepatic stellate cell activation by suppressing Sufu expression in non-alcoholic steatohepatitis [33], and so on. Interestingly, our study uncovered a novel function of miR-214 as a regulator of ferroptosis-driver genes *ACSL4*, *SLC38A1*, and *PRKAA2*. Unlike previous findings on miR-214, which focused on post-transcriptional downregulation, our study highlights its moonlighting effect in upregulating gene expressions. Although the exact target sites of miR-214 were not identified, to our knowledge, this is the first report of a nonconventional miRNA-mediated mechanism for regulating ferroptosis.

Our study explored the mechanism underlying miR-214-mediated upregulation of ferroptosis-driver genes. Our previous research demonstrated that miR-148a increases *ADH4* mRNA stability [34]; however, we found no effect of miR-214 on mRNA or primary RNA transcript stability for *ACSL4*, *SLC38A1*, and *PRKAA2*. Of note, the subcellular localization of miRNAs determines how they function [35], and we observed that most miR-214 localized to the nucleus in AML12 cells. Nuclear miRNAs have diverse functions, including direct modulation of cis-regulatory elements such as promoters and enhancers [36–38], regulation of the transcription factor activities [39, 40], interaction with chromatin modifiers like EZH2 [41], regulation of microprocessing machinery [42, 43], and remodeling of the chromatin architecture [44]. Our study revealed that miR-214 increased the enrichment levels of histone H3K29ac at the promoter regions of *ACSL4*, *SLC38A1*, and *PRKAA2*, indicating an activated chromatin state. Moreover, miR-214 also promoted RNA polymerase II loading to the promoter regions of target genes, suggesting that it facilitates the recruitment of the transcriptional apparatus. These results suggest that miR-214 activates ferroptosis-driver genes by increasing chromatin accessibility and RNAP II recruitment.

Previous studies have suggested that nuclear miRNAs can incorporate with either AGO1 or AGO2 protein to activate or repress gene transcription [45]. In our study, we observed that miR-214 selectively bound to AGO2 protein in the nucleus but not AGO1 protein. Co-transfection experiments revealed that the knockdown of AGO2 in primary hepatocytes abolished the increase of ACSL4 and PRKAA2 expressions induced by miR-214, indicating that miR-214 exerts a positive regulatory function by binding to AGO2 protein in the nucleus.

In this study, we demonstrated that chronic ethanol consumption leads to liver ferroptosis by causing the accumulation of labile irons and indirect inactivation of GPx4. Additionally, disruption of the methionine cycle and transsulfuration pathway results in decreased cysteine levels and, ultimately, GSH exhaustion. Furthermore, miR-214, which is upregulated in alcoholic hepatitis, increases ferroptosis by activating ferroptosis-driver genes (*ACSL4*, *PRKAA2*, and *SLC38A1*) through AGO2-dependent transcriptional mechanisms. These findings provide valuable insight for future therapeutic development targeting ferroptosis or miR-214 in alcohol-induced liver injury.

## Materials and methods

### Chemicals and reagents

The following chemicals and reagents were from commercial sources: DMEM, and penicillin–streptomycin, BasalMedia (Shanghai, China); FBS, GlutaMAX and William’s E Medium, Gibco (Grand Island, NY, USA); ethanol, Sigma-Aldrich (St. Louis, MO, USA); RSL3, Fer-1, Lip-1, Nec-1, Z-VAD, and CCK8 reagent, MedChem Express (Monmouth Junction, NJ, USA); miR-214-3p mimics, control mimics (miR-NC), miR-214 inhibitors (IH-214), and control inhibitors (IH-NC), GE Healthcare (Little Chalfont, Buckinghamshire, UK); siRNAs against *GPX4* (si-*GPX4*), siRNAs against *AGO1* (*si-AGO1*), siRNAs against *AGO2* (*si-AGO2*) and the negative control siRNAs (si-NC), GenePharma (Shanghai, China); primers, Tsingke (Beijing, China); ACSL4 antibody (Cat no: ab155282), PRKAA2 antibody (Cat no: ab97275), RNA plolymerase II ChIP-grade antibody (Cat no: ab264350), and H3K27ac ChIP-grade antibody (Cat no: ab4729), Abcam (Cambridge, MA, USA); β-Actin antibody (Cat no: 81115-1-RR) and goat-anti-mouse HRP antibody, Proteintech (Wuhan, Hubei, China); Rabbit IgG, Beyotime (Shanghai, China); AGO1 (Cat no: 03-249) and AGO2 (Cat no: 03-110) RIP antibody, Merck Millipore (Billerica, MA, USA).

### Animals

Six- to 8-week-old male C57BL/6J mice were randomly divided into two groups: the ethanol-fed group (*n* = 7) and the pair-fed group (*n* = 7). Mice in the ethanol-fed group were fed on a Lieber-DeCarli diet with 4% (v/w) ethanol (28% ethanol-derived calories) ad libitum or an isocaloric liquid diet for 8 weeks. On the last day, ethanol-fed mice were given a single gavage of ethanol (5 g/kg b.w., 31.25% ethanol), while the pair-fed mice were given an isocaloric gavage of dextrin maltose. The operator was blinded to the diets, which were prepared and numbered by the designer. None of the mice were excluded from the analysis. This animal experiment was approved by the Institutional Animal Care and Use Committee of Qingdao University.

### In silico analysis

The expression profiles of dysregulated miRNAs were retrieved from the Gene Expression Omnibus (GEO) datasets GSE59492. The list of validated ferroptosis-driver genes and ferroptosis inhibitor genes was downloaded from the FerrDb database (http://www.zhounan.org/ferrdb/legacy/index.html). The expression profiles of ferroptosis-related genes in alcoholic hepatitis patients and healthy individuals were retrieved from the Gene Expression Omnibus (GEO) datasets GSE28619.

### Primary hepatocytes isolation

Mouse primary hepatocytes were isolated according to a published protocol with some modifications [46]. An 8- to 10-week-old male C57/BL6J mouse was anesthetized by isopentane, and the liver was washed with a perfusion buffer containing 1×HBSS, 5 mM EGTA, and 55 mM Glucose. The hepatocytes were then dissociated by the digestion buffer containing 0.2 g/L collagenase IV and purified by 50% Percoll density separation. Finally, isolated hepatocytes were plated on collagen-coated cell culture plates.

### Cell culture and transfection

AML12 cells were obtained from the Cell Bank of the Chinese Academy of Sciences (Shanghai, China) and were cultured in DMEM containing 10% FBS. Mouse primary hepatocytes were cultured in William’s E medium supplemented with 10% FBS and 1% GlutaMAX. Cells were exposed to 0-, 50-, or 100-mM ethanol for 48 h, and the medium was changed every 24 h. To prevent ethanol evaporation, the cell incubators were equilibrated with 100 mM EtOH in the humidification pan.

The transfection of mimics, inhibitors, or siRNAs was performed according to the previous descriptions [47] with Lipofectamine™ RNAiMAX Transfection Reagent (Invitrogen, Carlsbad, CA, USA). The transfected cells were further incubated for another 48 h.

### Quantitative real-time PCR

Total RNA was extracted and purified with Trizol reagents (Takara, Tokyo, Japan), and 1 μg RNA was reverse transcribed into first cDNA using the PrimeScript™ RT reagent Kit (Takara). *q*RT-PCR assay was carried out using QuantiNova™ SYBR Green PCR reagent (Qiagen, Duesseldorf, Germany) and analyzed on the LightCycler® 480 System (Roche, Basel, Switzerland). The gene expression levels were normalized to the *ACTB* gene using the 2^−ΔΔCt^ method. Sequences of primers used in this study are listed in Supplementary Table S1.

### Immunoblotting

Cells were washed with ice-cold PBS and lysed with RIPA lysis buffer. Cell lysis was resolved by SDS-PAGE electrophoresis and transferred to PVDF membranes. Membranes were then blocked with 5% skimmed milk and incubated with antibodies against ACSL4, RPKAA2, AGO1, AGO2, and β-actin. Finally, an HRP-conjugated goat-anti-mouse/rabbit antibody was used, and target bands were detected by using the ECL reagent (GE Healthcare).

### Cell viability assay

Cell viability was determined by using the CCK8 reagent. Briefly, CCK8 solution was added to the culture medium, and cells were incubated at 37 °C for 1.5 h. The absorbance at 450 nm was measured using a microplate reader.

### The half-maximal inhibitory concentration (IC_50_) of RSL3

AML12 cells were treated with 0, 5, 10, 20, 30, 40, and 50 mM RSL3 for 24 h, then the cell inhibition rate was determined by CCK8 assay. The log values of RSL3 concentration and relative cell inhibition rates were then plotted on the *x*-axis and *y*-axis. IC_50_ value was calculated by using nonlinear regression analysis.

### RNA stability assay

Mouse primary hepatocytes were transfected with miR-214 mimics or control mimics for 24 h, and then actinomycin D (act-D) was added at the final concentration of 5 μg/ml. Cells were collected at 0, 2, 4, 8, and 16 h after act-D treatment. Relative mRNA or pre-mRNA levels were determined by *q*RT-PCR and normalized to *GAPDH*.

### Dual-luciferase reporter assay

The promoter sequence of ACSL4, PRKAA2, and SLC38A1 was synthesized and cloned to pGL3-basic vectors (Tsingke, Beijing, China), respectively. Then, 500 ng recombinant plasmids, 5 ng pRL-SV40, and miR-214 mimics or inhibitors were transfected into HEK-293FT cells. The luciferase activities were measured by Dual-Luciferase Reporter Assay Kit (Vazyme, Nanjing, China).

### Chromatin immunoprecipitation (ChIP)

ChIP assay was performed as previously described with some modifications [48]. Briefly, primary hepatocytes were cross-linked with 1% formaldehyde and quenched by 125 mM glycine. Then cell nucleus was fractioned with buffer containing 5 mM PIPES (PH = 8.0), 85 mM KCl, and 0.5% NP-40 and lysed in buffer containing 1% SDS. Chromatin DNA was sonicated and incubated with RNA polymerase II antibody, H3K27ac antibody, and H3K4me1 antibody. Protein A/G magnet beads were used to pull down the bound chromatin DNA fragments. The enrichment of *ACSL4*, *PRKKA2*, and *SLC38A1* promoter DNA was determined by quantitative PCR.

### Cell fraction assay

First, the cytoplasmic fraction and nuclear fraction were separated as described previously [34]. Then, total RNA was extracted, and 500 ng RNA was reverse transcribed with miR-214 specific stem-loop primer by using the Revert Aid First Strand cDNA Synthesis Kit (Thermo Scientific, Waltham, MA, USA). After *q*RT-PCR analysis, the expression ratio of miR-214 in cytoplasm and nuclei was calculated using the ΔCt method. GAPDH was used as a cytoplasmic marker, while U1 was a nuclear marker.

### RNA immunoprecipitation (RIP)

RIP assay was conducted using an RNA-chromatin immunoprecipitation protocol. The cytoplasmic fraction and the nuclear fraction were isolated from the cross-linked primary hepatocytes as the ChIP assay described. Then AGO1 antibody, AGO2 antibody, and control IgG were added to the cytoplasmic fraction and the nuclear fraction, respectively. The immune complexes were pulled down by Protein A/G magnet beads and then eluted by the addition of an Elution Buffer containing 0.1 M NaHCO3 and 1% SDS. After reverse cross-linking, the enrichment levels of miR-214 were determined by *q*RT-PCR.

### Statistical analysis

Results were shown as mean ± SD from at least three independent experiments. Student’s *t*-test was applied to analyze the enrichment differences between the miR-214 mimics-transfected group and the control group in the ChIP assay. One-way ANOVA analysis followed by Dunnetts’ multiple comparisons test was performed between multiple groups. *P* < 0.05 was considered statistically significant. In addition, Pearson correlation analysis was conducted to determine the expression correlations between miR-214 and its target genes. The correlation coefficients value *r* > 0.2 was considered to be correlated. GraphPad Prism 7.00 software was used to perform all statistical analyses.

### Supplementary information

Supplemental Material

Original Data File

## Data Availability

The data that support the findings of this study are available upon reasonable request from the corresponding author.
