# Supplementary material for: Ferroptosis contributes to ethanol-induced hepatic cell death via labile iron accumulation and GPx4 inactivation
Source: Cell Death Discov. 2023 Aug 25;9:311. doi: 10.1038/s41420-023-01608-6 (PMC10457354; doi:10.1038/s41420-023-01608-6)
Supplement: Supplementary file 1 — Supplemental Material [file 41420_2023_1608_MOESM1_ESM.docx]

**Ferroptosis contributes to ethanol-induced hepatic cell death via labile iron accumulation and GPx4 inactivation**

Jiao Luo^1,2^, Ge Song^1,2^, Ningning Chen^1,2^, Mengyue Xie^1^, Xuan Niu^1^, Shuyue Zhou^1^, Yanan Ji^1^, Xiaoxiao Zhu^1^, Wanli Ma^1^, Qianqian Zhang^1^, Dianke Yu^1,3^

^1^School of Public Health, Qingdao University, Qingdao, China

^2^These authors contributed equally: Jiao Luo, Ge Song, and Ningning Chen.

^3^Corresponding author:

Dianke Yu, Ph.D.

Email: [dianke.yu@qdu.edu.cn](mailto:dianke.yu@qdu.edu.cn)


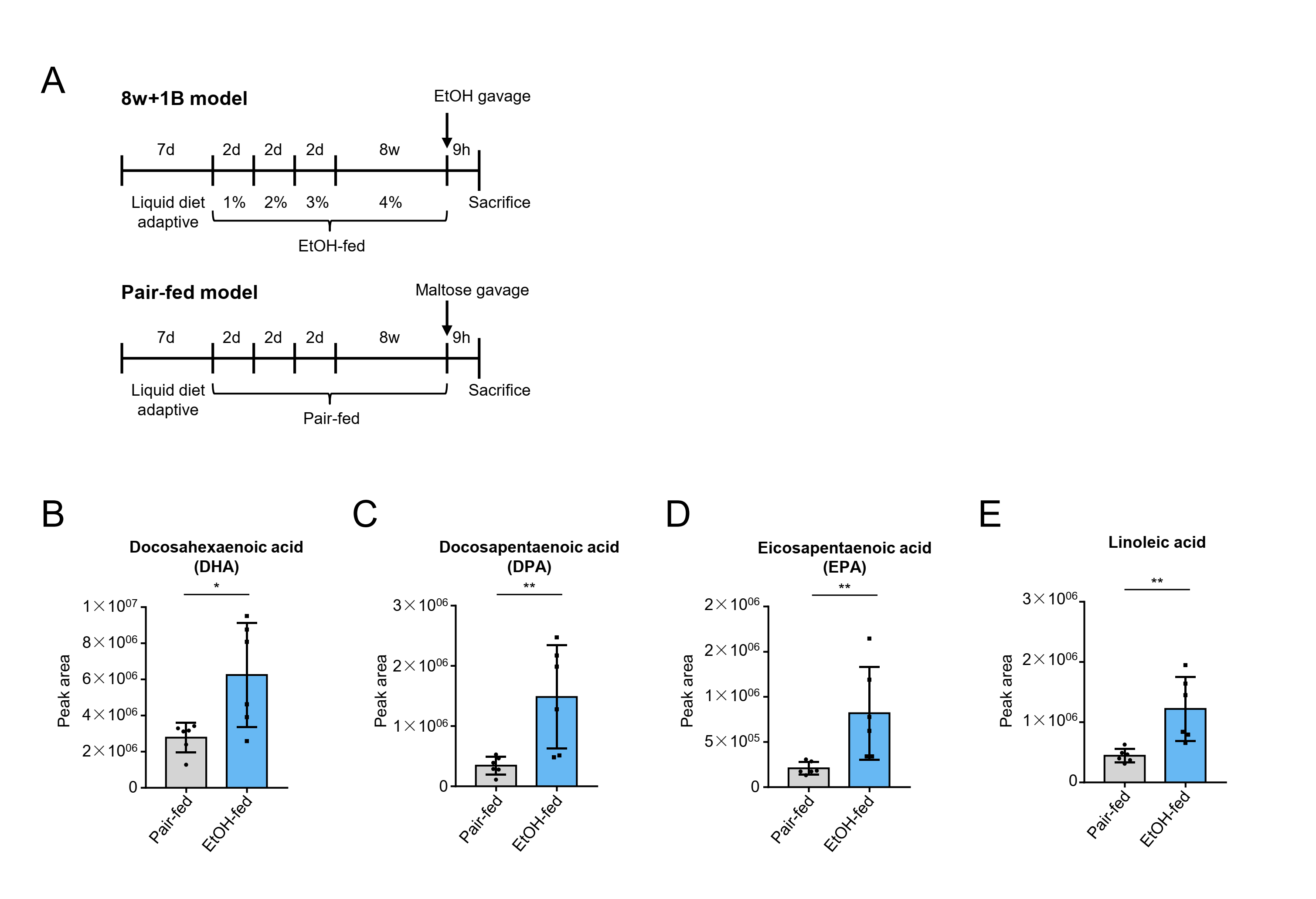


Fig. S1. (A) Figure illustration of the establishment of ALD mice model by long-term ethanol feeding. 8- to 10-week-old male C57BL/6 mice were first adapted to Lieber-DeCarli liquid diet for 1 week, and then adapted to 1%-, 2%-, and 3%-ethanol (w/w) containing Lieber-DeCarli liquid diet for 2 days, respectively. Then mice were allowed free access to the liquid diet containing 4% (w/w) ethanol for 8 weeks. At the last day, ethanol-fed mice received a single dose of ethanol (5 g/kg body weight) by gavage at the early morning and sacrificed 9 h later. Pair-fed mice were fed with the Lieber-DeCarli control diet, followed by isocaloric dextrin-maltose gavage. (B-E) Hepatic levels of polyunsaturated fatty acids (PUFAs) DHA (B), DPA (C), EPA (D), and Linoleic acid (E) from ethanol-fed mice and pair-fed mice. **p* < 0.05, ***p* < 0.01, and ****p* < 0.001 as indicated, respectively.


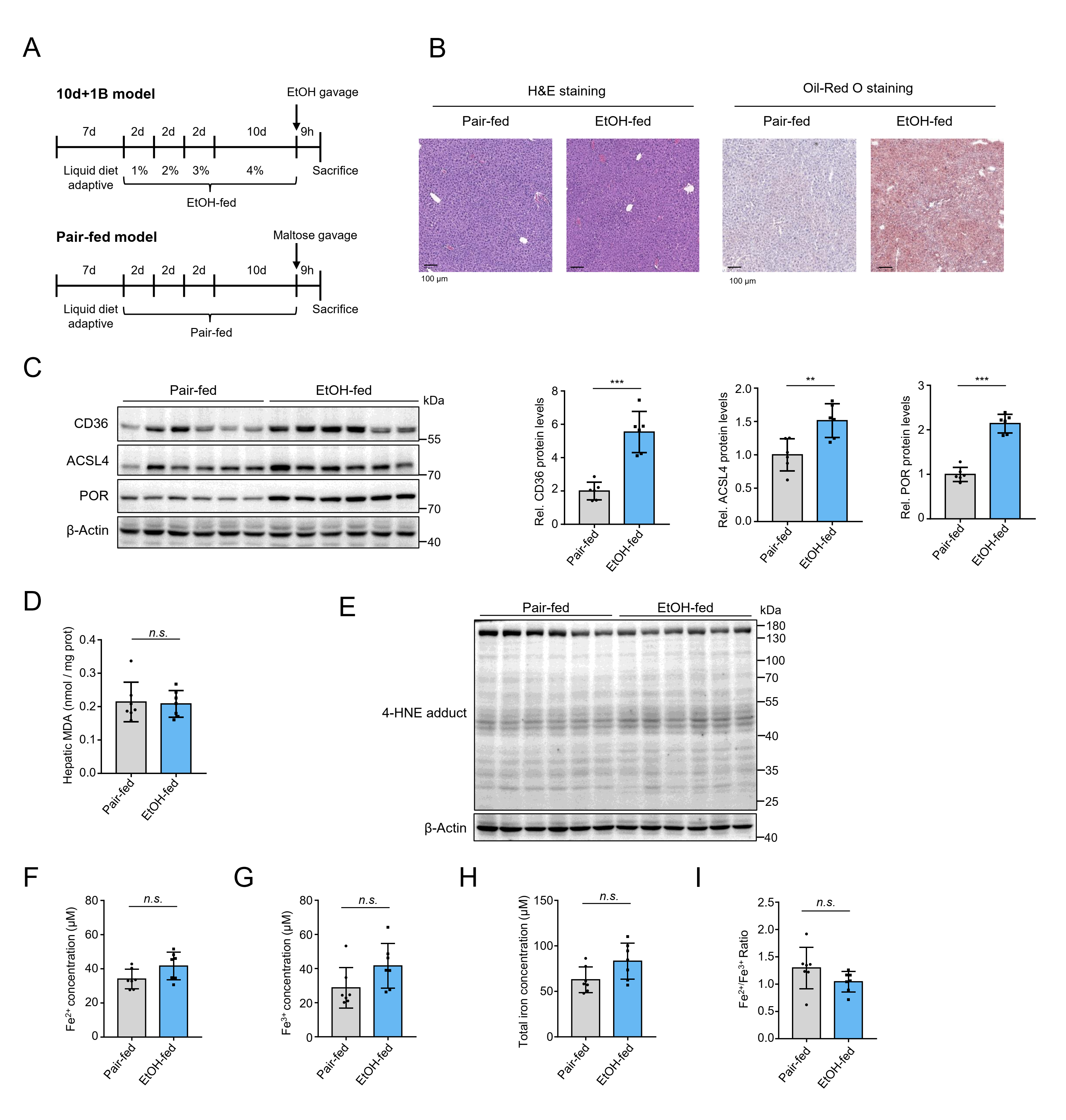


Fig. S2. (A) Figure illustration of the establishment of E10d+1B mice model. (B) Liver H&E staining and Oil-Red O staining of E10d+1B mice and pair-fed mice. (C) Immunoblotting analysis of the expressions of CD36, LPCAT3, ACSL4, and POR in the liver of E10d+1B mice and pair-fed mice. (D) Hepatic MDA levels of E10d+1B mice and pair-fed mice. (E) 4-HNE adduct levels in the liver of E10d+1B mice and pair-fed mice analyzed by immunoblotting. (F-H) Liver concentrations of ferrous irons (F), ferric irons (G), and total irons (H) in E10d+1B mice and pair-fed mice. (I) The ratios of ferrous irons to ferric irons in the liver of E10d+1B mice and pair-fed mice. *n.s.*, not significant; **p* < 0.05, ***p* < 0.01, ****p* < 0.001 as indicated.


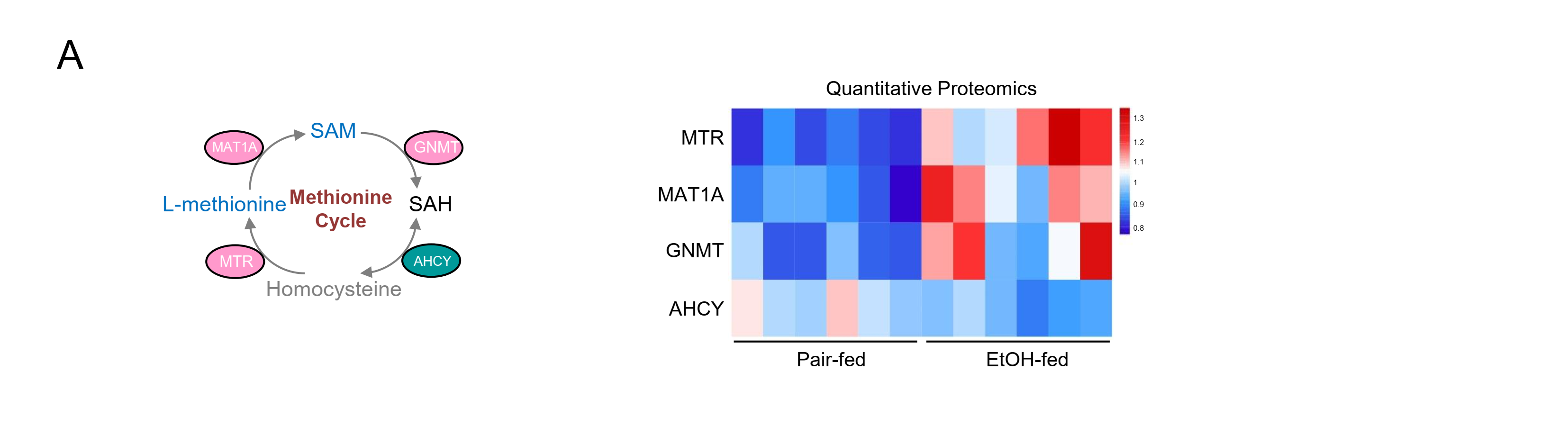


Fig. S3. (A) Heatmap showing MAT1A, GNMT, MTR, and AHCY protein expressions. The expression profiles were retrieved from our TMT proteomics data.


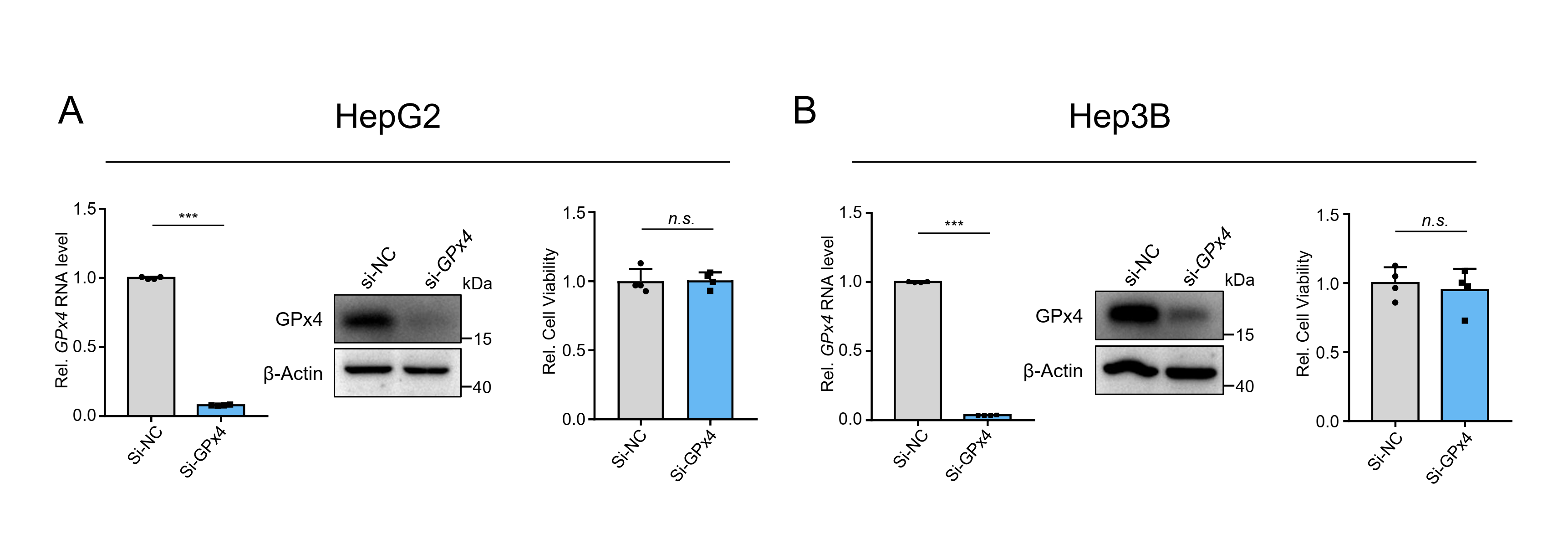


Fig. S4. (A) Cell viability of HepG2 cells transfected with GPX4 siRNAs (50 nM) for 48 h. (B) Cell viability of Hep3B cells transfected with GPX4 siRNAs (50 nM) for 48 h. *n.s.*, not significant; **p* < 0.05, ***p* < 0.01, ****p* < 0.001 as indicated.


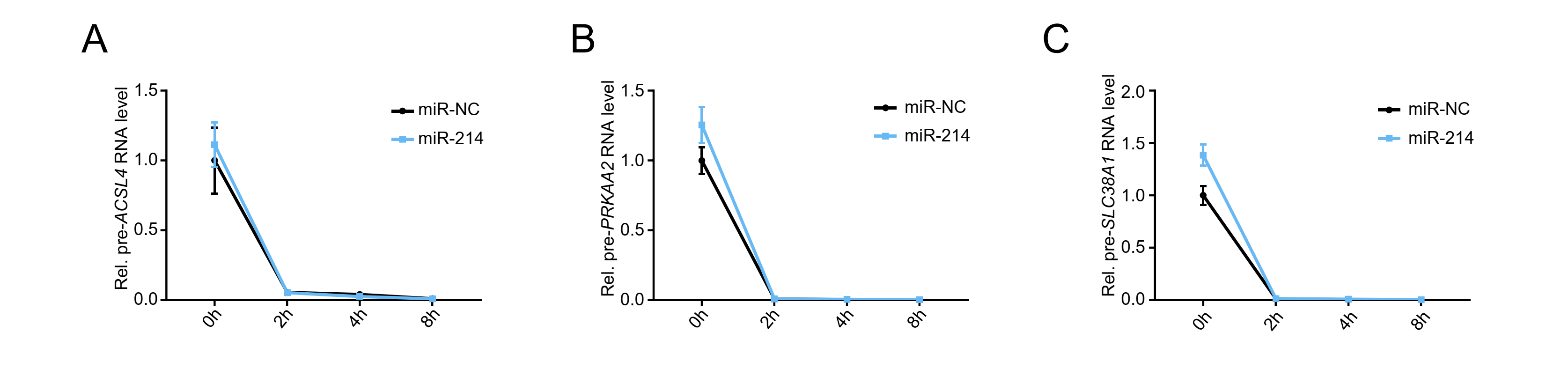


Fig. S5. Effects of miR-214 on the primary RNA stability of *ACSL4* (A), *PRKAA2* (B), and *SLC38A1* (C). Primary hepatocytes were transfected with miR-214 mimics or control mimics at the final concentration of 20 nM. After 24 h incubation, cells were treated with 5 μg/ml actinomycin D for additional 2, 4, 8, 12, and 16 h, respectively. Relative RNA levels were measured by *q*RT-PCR and normalized by *GAPDH*.

**Table S1.** The sequence conservation of top25 up-regulated miRNAs in AH patient livers^*^

| **miRNA** | **Human sequence** | **Mouse sequence** | **conservation** | **Log_2_FC** |
| --- | --- | --- | --- | --- |
| hsa-miR-182-5p | uuuggcaaugguagaacucacacu | uuuggcaaugguagaacucacaccg | Seed conserved | 2.43 |
| hsa-miR-4521 | gcuaaggaaguccugugcucag | N.A. | Not conserved | 2.02 |
| hsa-miR-21-5p | uagcuuaucagacugauguuga | uagcuuaucagacugauguuga | Conserved | 2.01 |
| hsa-miR-503-5p | uagcagcgggaacaguucugcag | uagcagcgggaacaguacugcag | Seed conserved | 1.97 |
| hsa-miR-127 | ucggauccgucugagcuuggcu | ucggauccgucugagcuuggcu | Conserved | 1.86 |
| hsa-miR-214-3p | acagcaggcacagacaggcagu | acagcaggcacagacaggcagu | Conserved | 1.82 |
| hsa-miR-146b | ugagaacugaauuccauaggcug | ugagaacugaauuccauaggcu | Seed conserved | 1.79 |
| hsa-miR-720 | no mature sequence | no mature sequence | Not conserved | 1.76 |
| hsa-miR-132-3p | uaacagucuacagccauggucg | uaacagucuacagccauggucg | Conserved | 1.68 |
| hsa-miR-3201 | gggauaugaagaaaaau | N.A. | Not conserved | 1.68 |
| hsa-miR-224-3p | aaaauggugcccuagugacuaca | aaauggugcccuagugacuaca | Seed conserved | 1.63 |
| hsa-miR-3178 | ggggcgcggccggaucg | N.A. | Not conserved | 1.62 |
| hsa-miR-432-5p | ucuuggaguaggucauugggugg | ucuuggaguagaucagugggcag | Seed conserved | 1.59 |
| hsa-miR-3195 | cgcgccgggcccggguu | N.A. | Not conserved | 1.45 |
| hsa-miR-3180-3p | uggggcggagcuuccggaggcc | N.A. | Not conserved | 1.42 |
| hsa-miR-339-3p | ugagcgccucgacgacagagccg | ugagcgccucggcgacagagccg | Seed conserved | 1.42 |
| hsa-miR-3128 | ucuggcaaguaaaaaacucucau | N.A. | Not conserved | 1.38 |
| hsa-miR-222-3p | agcuacaucuggcuacugggu | agcuacaucuggcuacugggucu | Conserved | 1.33 |
| hsa-miR-652-3p | aauggcgccacuaggguugug | aauggcgccacuaggguugug | Conserved | 1.33 |
| hsa-miR-4485-3p | uaacggccgcgguacccuaa | N.A. | Not conserved | 1.32 |
| hsa-miR-3180 | uggggcggagcuuccggag | N.A. | Not conserved | 1.28 |
| hsa-miR-221-3p | agcuacauugucugcuggguuuc | agcuacauugucugcuggguuuc | Conserved | 1.27 |
| hsa-miR-99b-5p | cacccguagaaccgaccuugcg | cacccguagaaccgaccuugcg | Conserved | 1.22 |
| hsa-miR-214-5p | ugccugucuacacuugcugugc | ugccugucuacacuugcugugc | Conserved | 1.2 |
| hsa-miR-34a-5p | uggcagugucuuagcugguugu | uggcagugucuuagcugguugu | Conserved | 1.17 |

AH, alcoholic hepatitis; N.A., non-available; *Expression profiles were retrieved from GSE59492 dataset.

**Table S2**. Significantly upregulated ferroptosis-driver genes in alcoholic hepatitis patients

| **Symbol** | **Name** | **P value** | **Log_2_FC** |
| --- | --- | --- | --- |
| ACSL4 | Acyl-CoA synthetase long chain family member 4 | 3.44E-07 | 3.51 |
| PRKAA2 | Protein kinase AMP-activated catalytic subunit alpha 2 | 1.32E-08 | 2.53 |
| SLC38A1 | Solute carrier family 38 member 1 | 2.42E-08 | 2.93 |

**Table S3**. qPCR primers for this study

| **Name** | **Forward sequence (5’–3’)** | **Forward sequence (5’–3’)** |
| --- | --- | --- |
| **qRT-PCR primers** | | |
| m*Acsl4* | CCTTTGGCTCATGTGCTGGAAC | GCCATAAGTGTGGGTTTCAGTAC |
| m*Prkaa2* | CTGAAGCCAGAGAATGTGCTGC | GAGATGACCTCAGGTGCTGCAT |
| m*Slc38a1* | TACCAGAGCACAGGCGACATTC | ATGGCGGCACAGGTGGAACTTT |
| miR-214 stem-loop primers | |  |
| GTCGTATCCAGTGCGTGTCGTGGAGTCGGCAAT TGCACTGGATACGACACTGCCT | | |
| miR-214 | GGACAGCAGGCACAGACAG | CAGTGCGTGTCGTGGAGT |
| *mGpx4* | CCTCTGCTGCAAGAGCCTCCC | CTTATCCAGGCAGACCATGTGC |
| *hGPx4* | ACAAGAACGGCTGCGTGGTGAA | GCCACACACTTGTGGAGCTAGA |
| *mU6* | CTCGCTTCGGCAGCACA | AACGCTTCACGAATTTGCGT |
| *mActb* | CATTGCTGACAGGATGCAGAAGG | TGCTGGAAGGTGGACAGTGAGG |
| *hACTB* | CACCATTGGCAATGAGCGGTTC | AGGTCTTTGCGGATGTCCACGT |
| *mGapdh* | CATCACTGCCACCCAGAAGACTG | ATGCCAGTGAGCTTCCCGTTCAG |
| **ChIP-PCR primers** | | |
| Acsl4 | GCCTCCGCTCACTAAGGAC | GAAGCAGCCCTCCTCCTTT |
| Prkaa2 | ACTCTTCACTTTGCCGAAGG | GAACATGGCTGAGAAGCAGA |
| Slc38a1 | GTTGAGCTGTGTGCGGTAGA | TTCCTGGATGCTCGGTGT |

**Table S4**. siRNA sequences used in this study

| **Name** | **Forward sequence (5’–3’)** | **Reverse sequence (5’–3’)** |
| --- | --- | --- |
| si-m*Ago1*-1# | GCGGGAAACAGUUCUACAATT | UUGUAGAACUGUUUCCCGCTT |
| si-m*Ago1*-2# | CAGCCAUGCUUCUGCAAAUTT | AUUUGCAGAAGCAUGGCUGTT |
| si-m*Ago2*-1# | GGAGAGUGAACAGGGAAAUTT | AUUUCCCUGUUCACUCUCCTT |
| si-m*Ago2*-2# | GCUGGACAGAGAUGUAUAATT | UUAUACAUCUCUGUCCAGCTT |
| si-m*GPx4*-1# | GGAGCCAGGAAGUAAUCAATT | UUGAUUACUUCCUGGCUCCTT |
| si-m*GPx4*-2# | GCUGGGAAAUGCCAUCAAATT | UUUGAUGGCAUUUCCCAGCTT |
