# Supplementary material for: Ferroptosis contributes to ethanol-induced hepatic cell death via labile iron accumulation and GPx4 inactivation
Source: Cell Death Discov. 2023 Aug 25;9:311. doi: 10.1038/s41420-023-01608-6 (PMC10457354; doi:10.1038/s41420-023-01608-6)
Supplement: Supplementary file 2 — Original Data File [file 41420_2023_1608_MOESM2_ESM.pptx]

## Slide 1
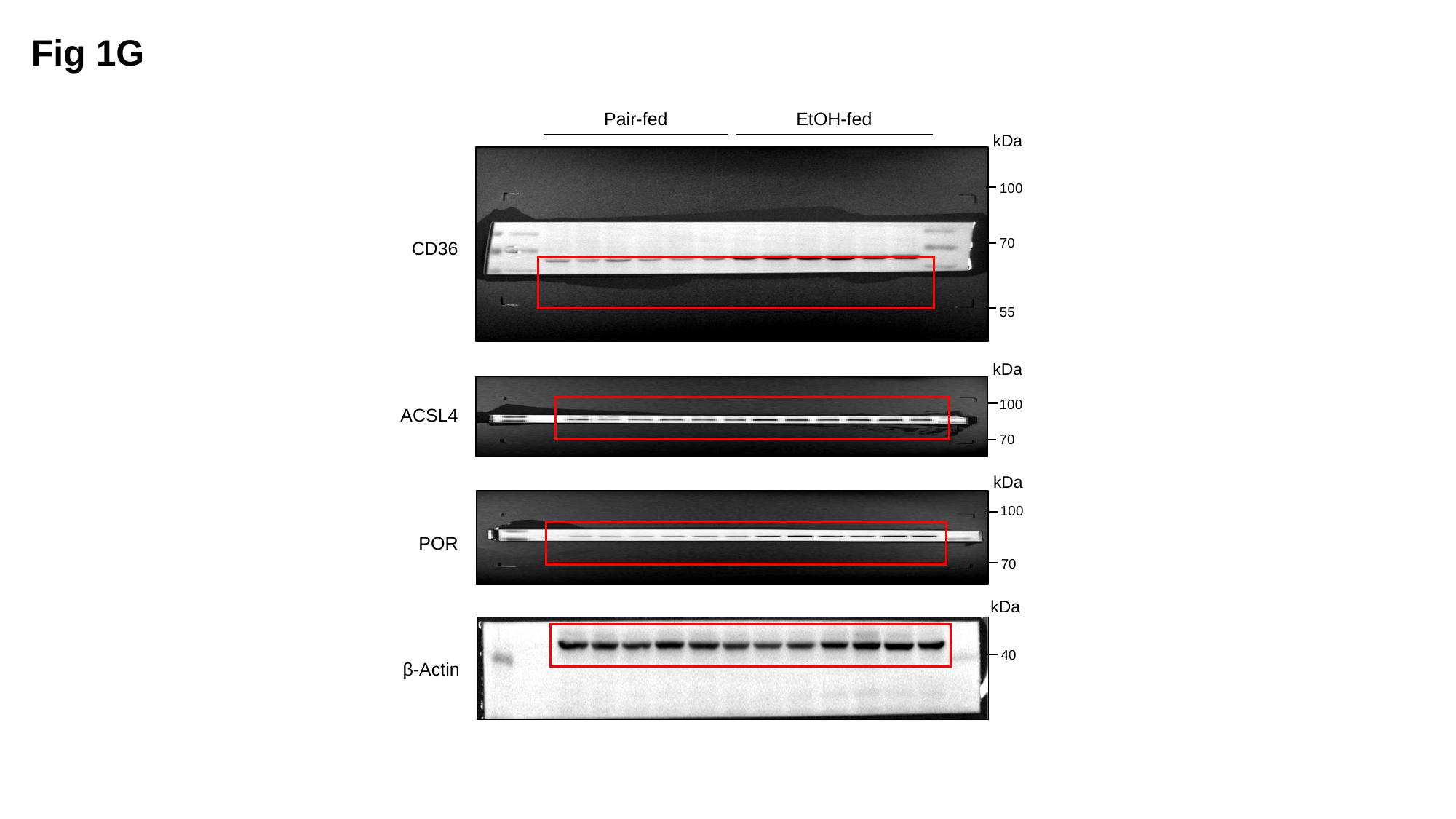

Fig 1G
Pair-fed
EtOH-fed
kDa
100
70
CD36
55
kDa
100
ACSL4
70
kDa
100
POR
70
kDa
40
β-Actin

## Slide 2
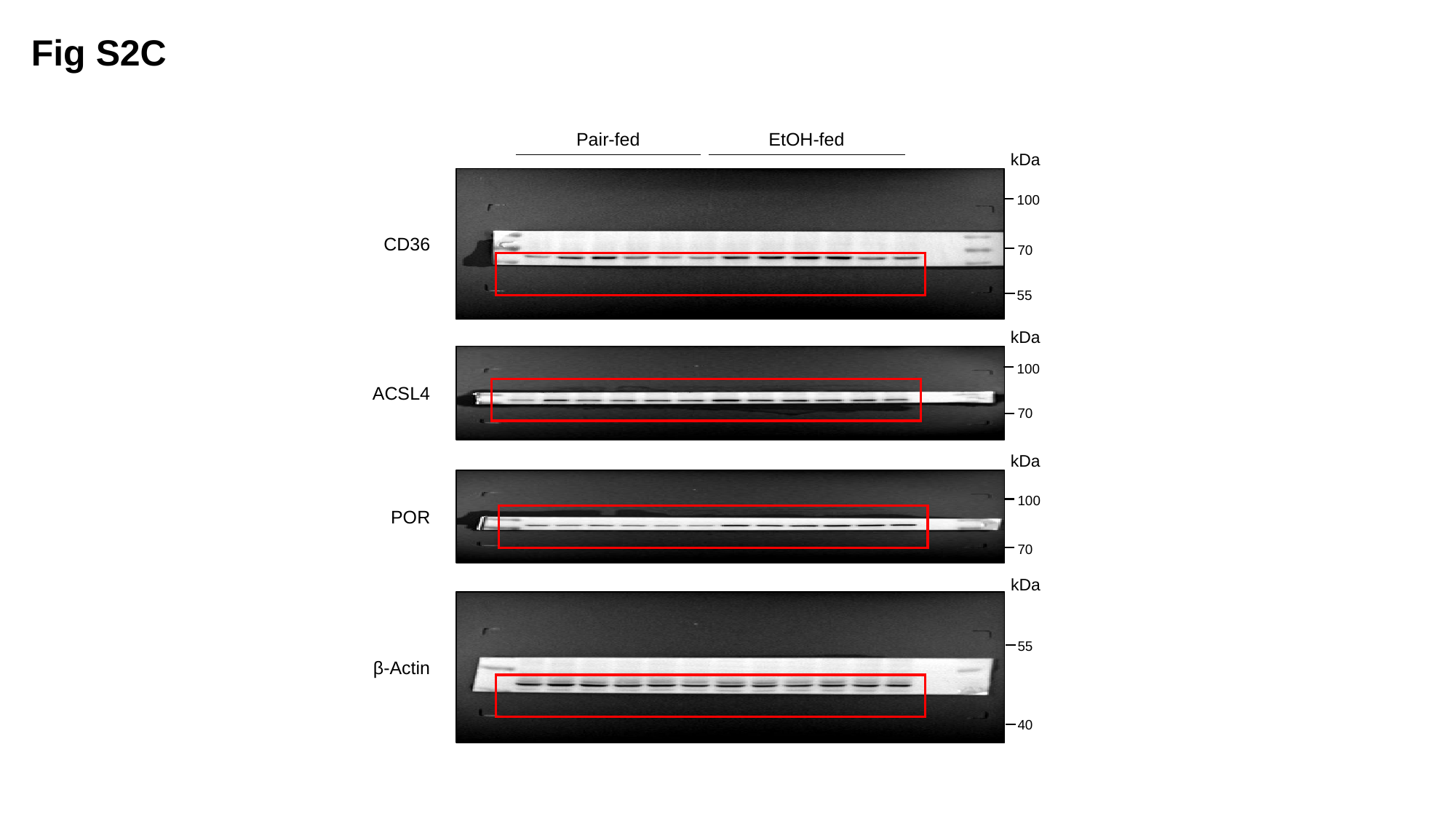

Fig S2C
Pair-fed
EtOH-fed
kDa
100
CD36
70
55
kDa
100
ACSL4
70
kDa
100
POR
70
kDa
55
β-Actin
40

## Slide 3
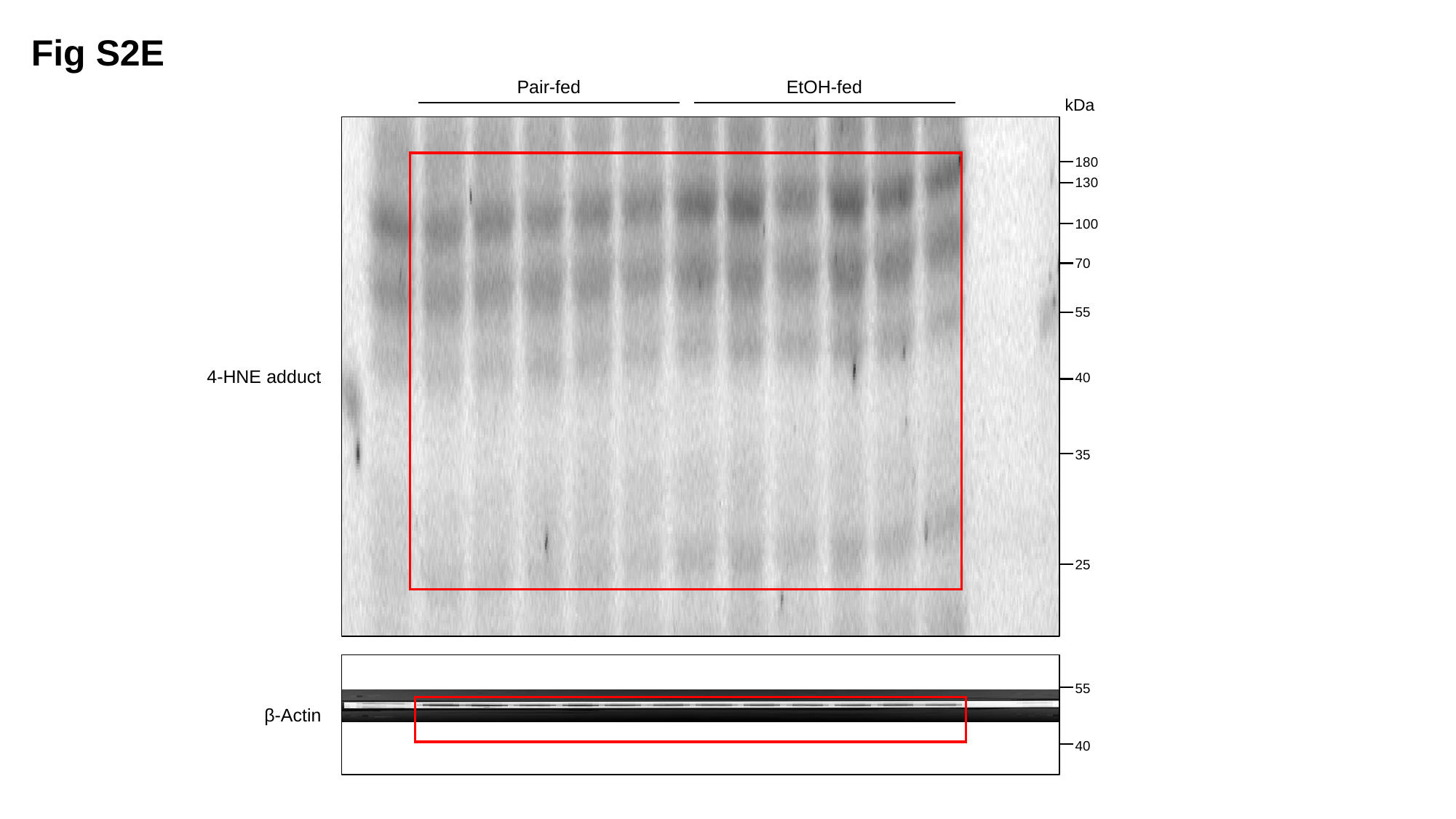

Fig S2E
Pair-fed
EtOH-fed
kDa
180
130
100
70
55
4-HNE adduct
40
35
25
55
β-Actin
40

## Slide 4
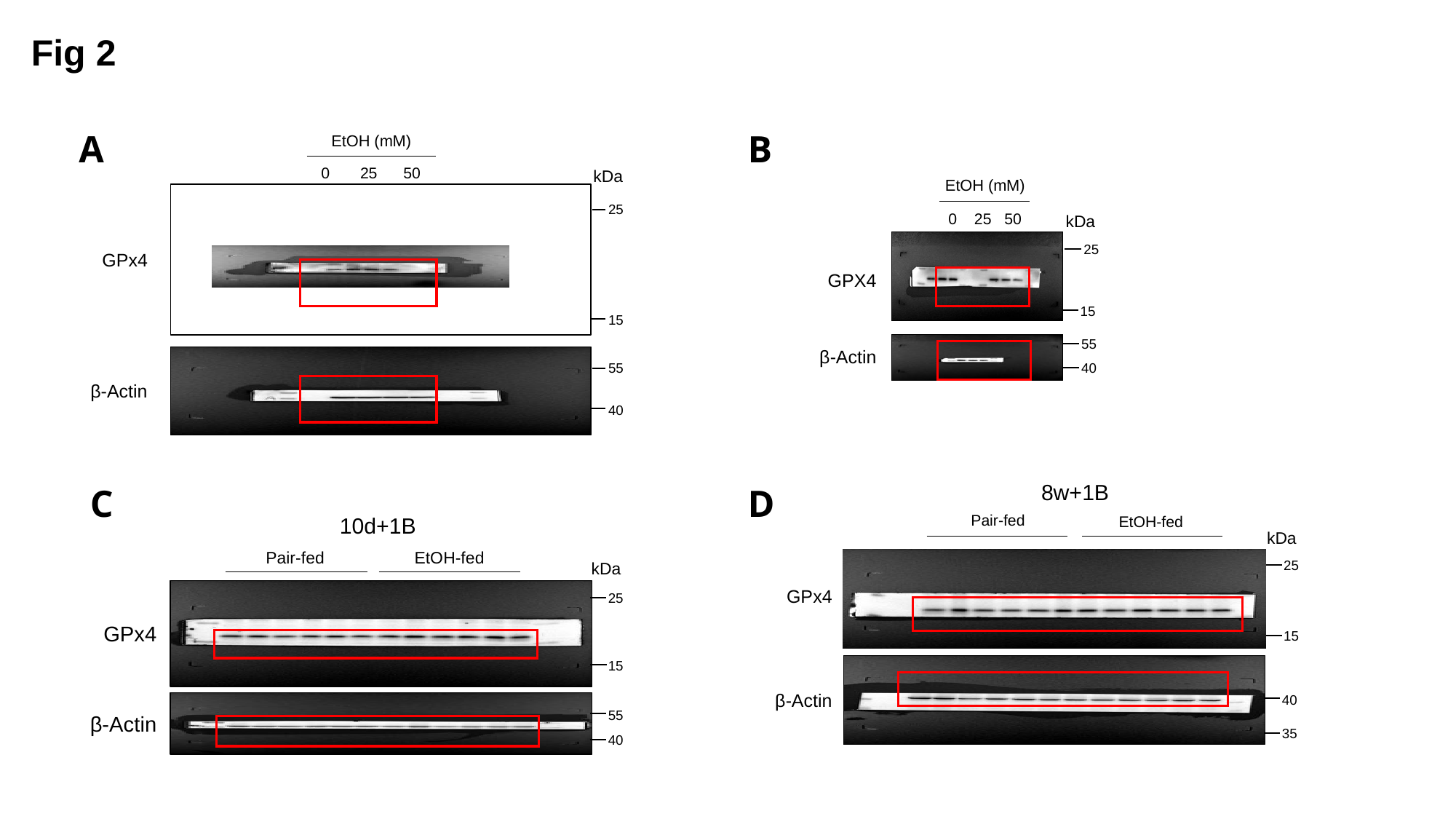

Fig 2
A
B
EtOH (mM)
 0 25 50
kDa
25
GPx4
15
55
β-Actin
40
EtOH (mM)
 0 25 50
kDa
25
GPX4
15
55
β-Actin
40
8w+1B
C
D
Pair-fed
EtOH-fed
10d+1B
kDa
EtOH-fed
Pair-fed
25
kDa
GPx4
25
GPx4
15
15
β-Actin
40
55
β-Actin
35
40

## Slide 5
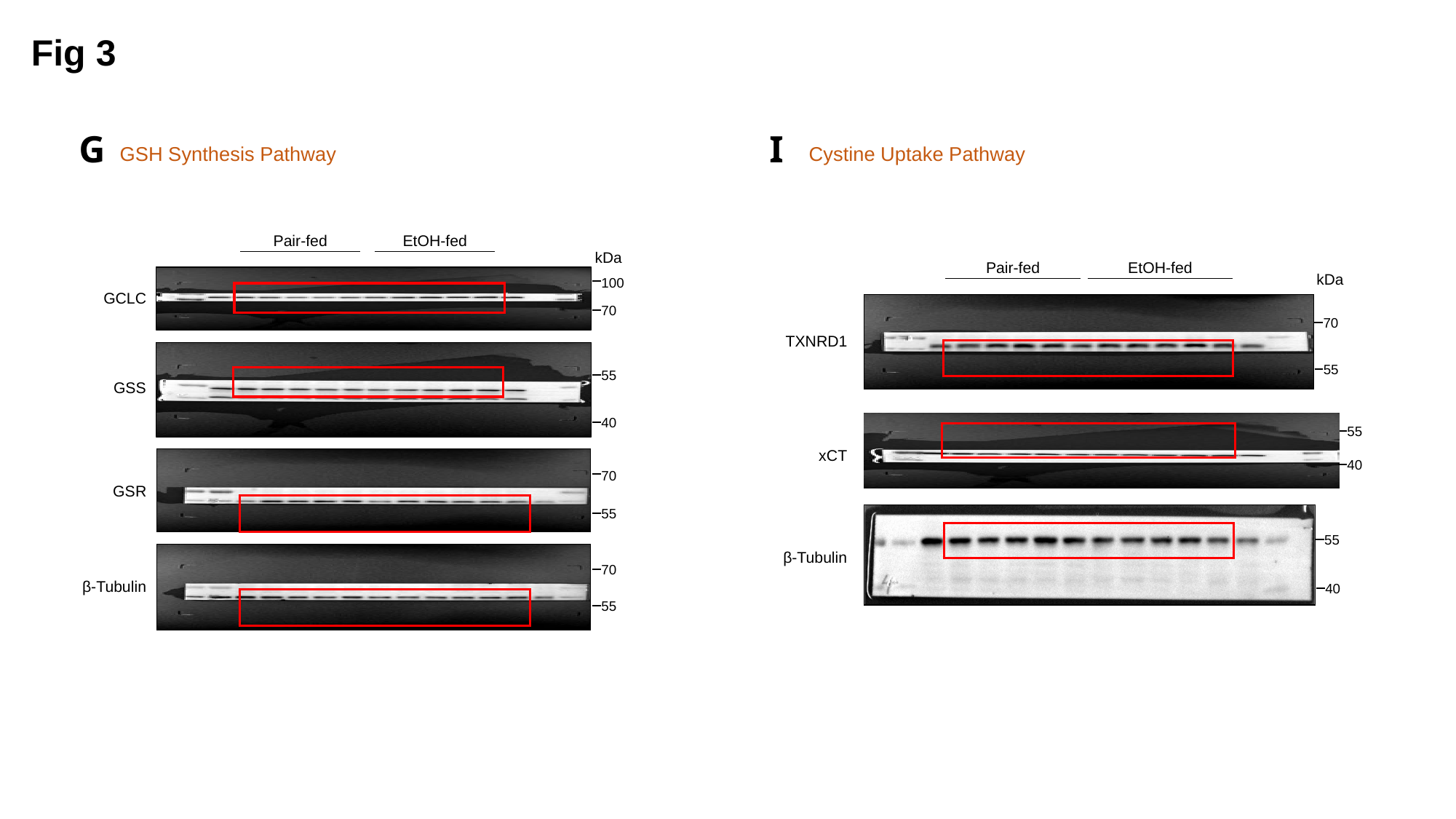

Fig 3
G
I
GSH Synthesis Pathway
Cystine Uptake Pathway
Pair-fed
EtOH-fed
kDa
EtOH-fed
Pair-fed
kDa
100
GCLC
70
70
TXNRD1
55
55
GSS
40
55
xCT
40
70
GSR
55
55
β-Tubulin
70
β-Tubulin
40
55

## Slide 6
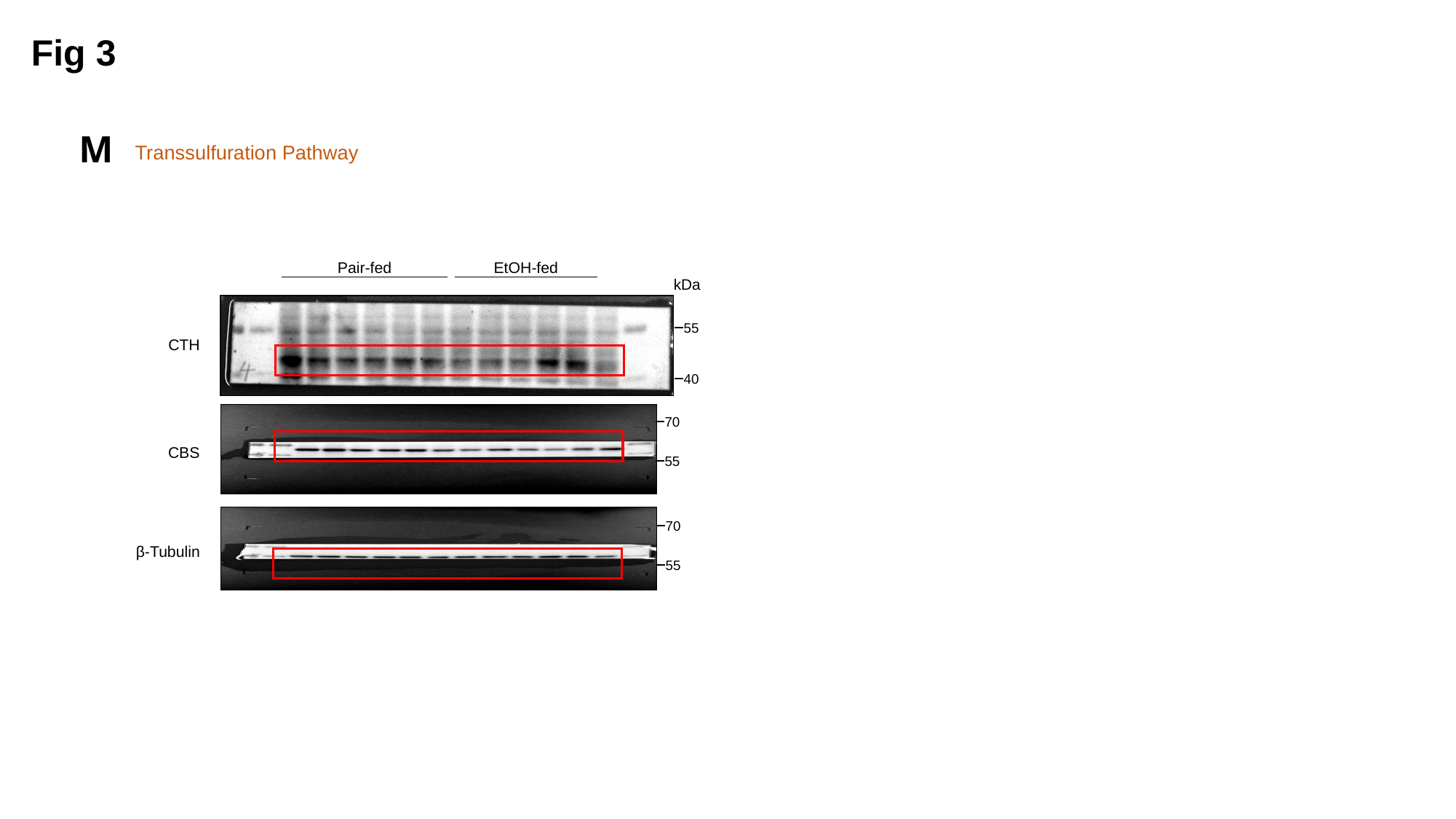

Fig 3
M
Transsulfuration Pathway
EtOH-fed
Pair-fed
kDa
55
CTH
40
70
CBS
55
70
β-Tubulin
55

## Slide 7
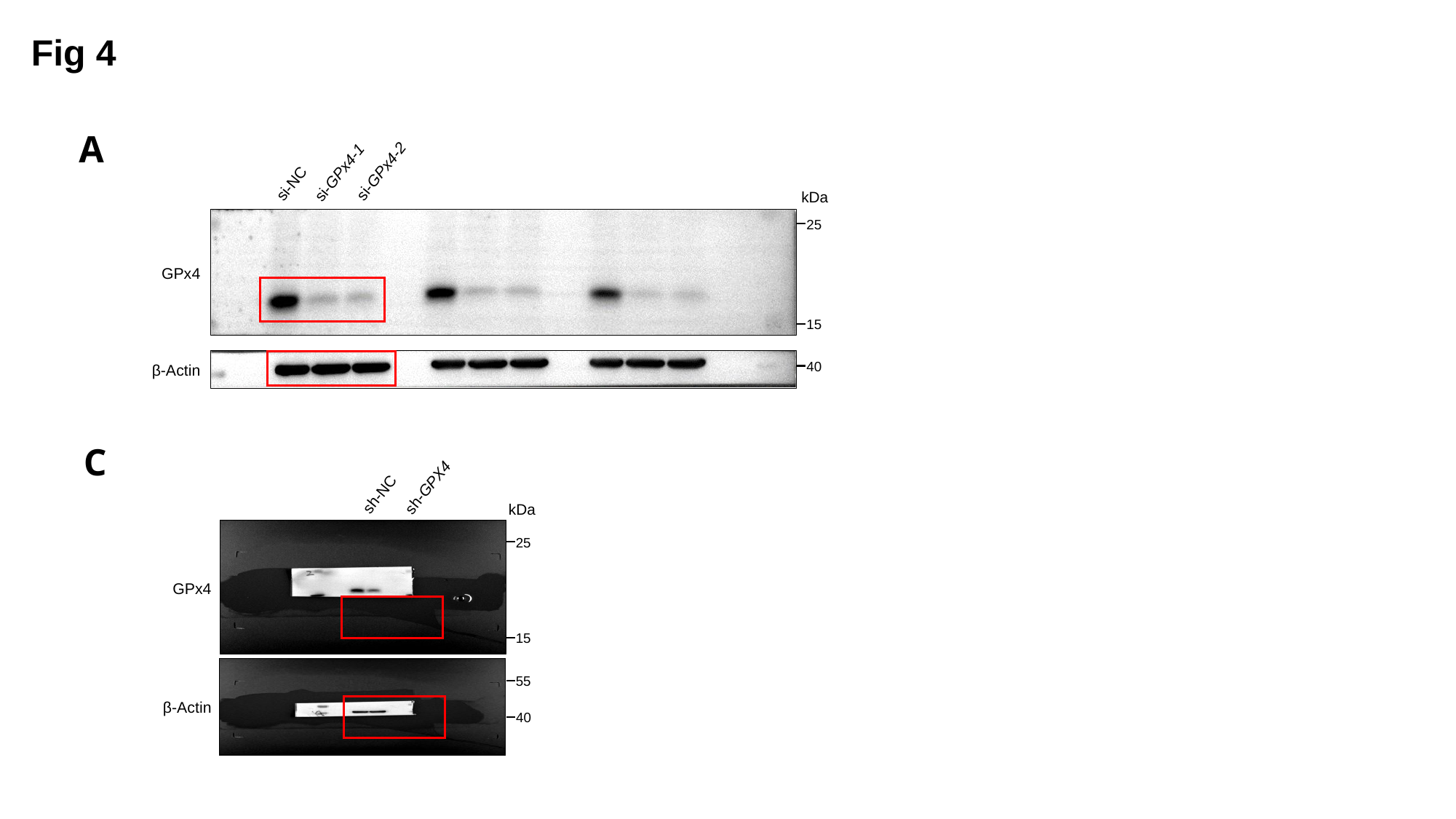

Fig 4
A
si-GPx4-2
si-GPx4-1
si-NC
kDa
25
GPx4
15
40
β-Actin
C
sh-GPX4
sh-NC
kDa
25
GPx4
15
55
β-Actin
40

## Slide 8
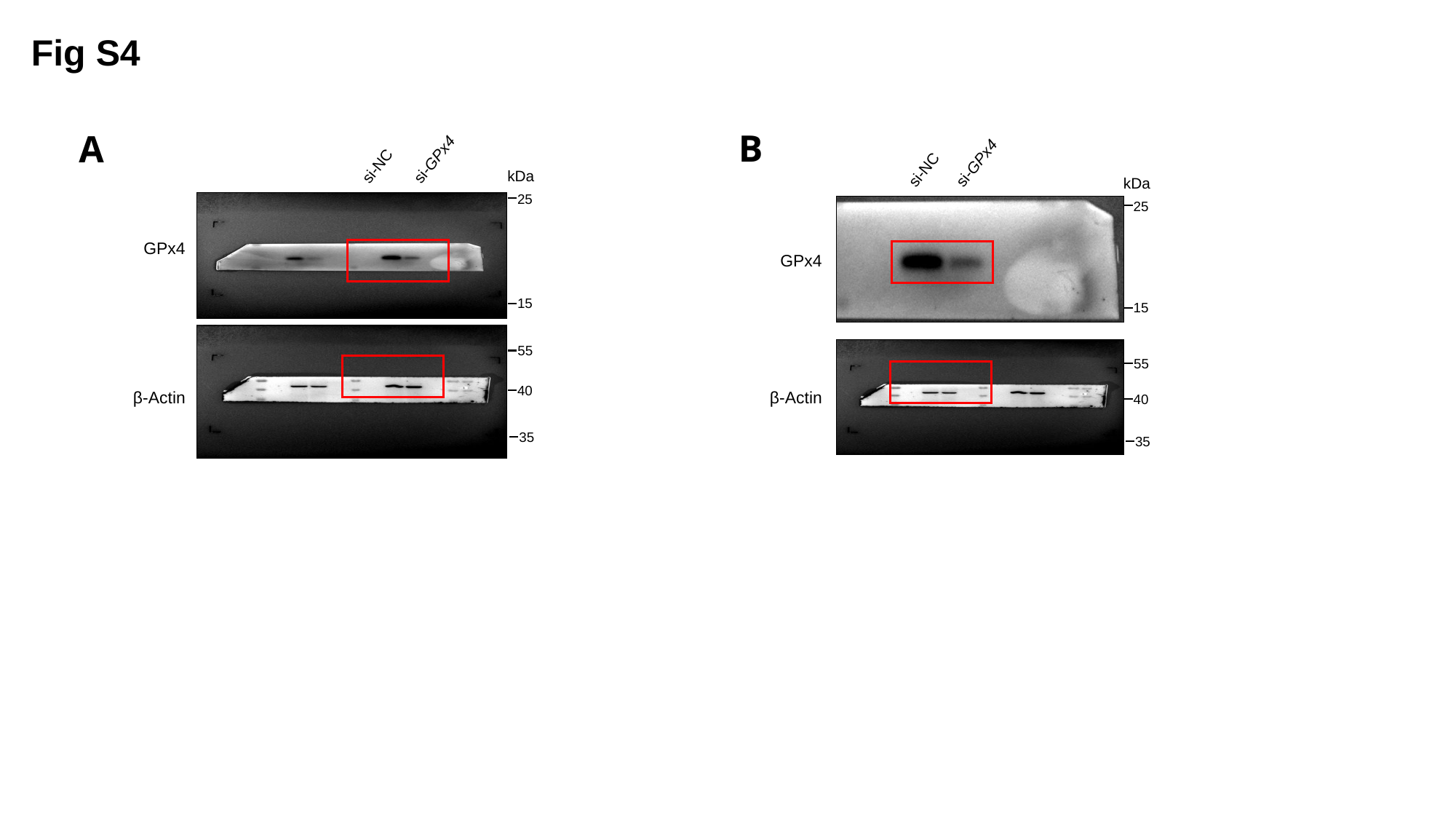

Fig S4
B
A
si-GPx4
si-GPx4
si-NC
si-NC
kDa
kDa
25
25
GPx4
GPx4
15
15
55
55
40
β-Actin
β-Actin
40
35
35

## Slide 9
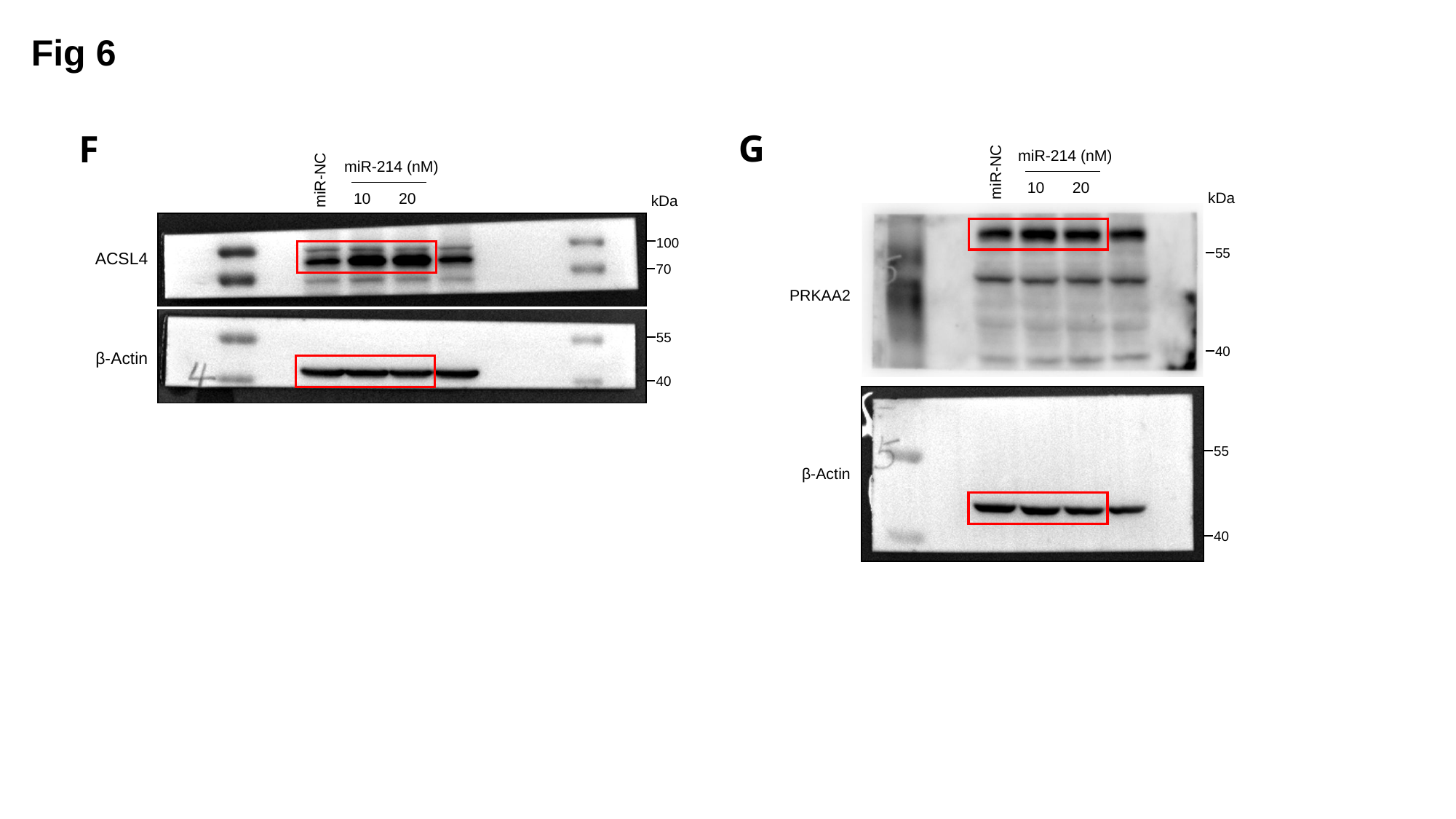

Fig 6
miR-214 (nM)
miR-NC
10
20
G
F
miR-214 (nM)
miR-NC
10
20
kDa
kDa
100
55
ACSL4
70
PRKAA2
55
40
β-Actin
40
55
β-Actin
40

## Slide 10
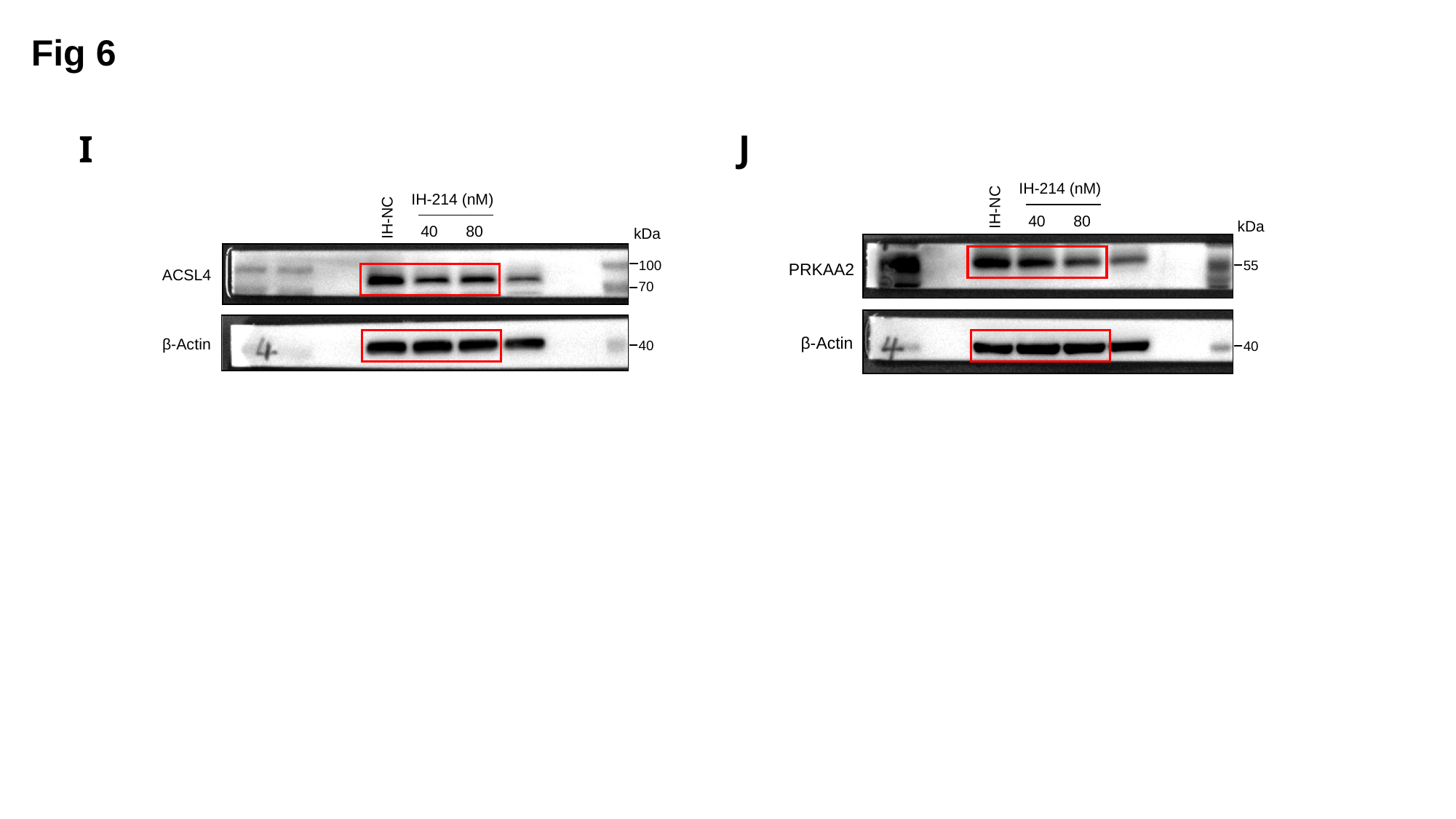

Fig 6
J
I
IH-214 (nM)
IH-NC
40
80
kDa
55
PRKAA2
β-Actin
40
IH-214 (nM)
IH-NC
40
80
kDa
100
ACSL4
70
β-Actin
40

## Slide 11
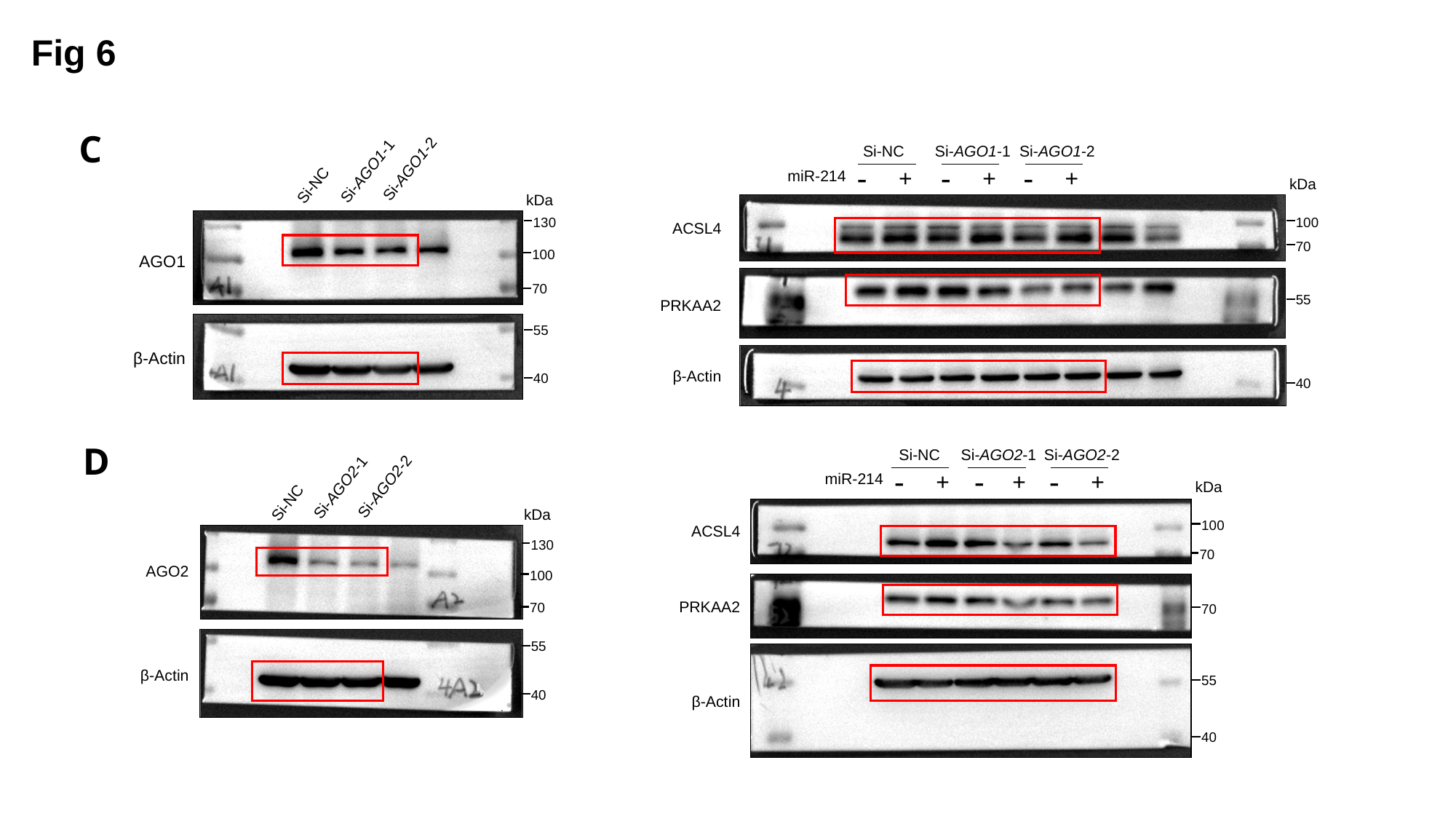

Fig 6
C
Si-NC
Si-AGO1-1
Si-AGO1-2
-
-
-
+
+
+
miR-214
Si-AGO1-2
Si-AGO1-1
Si-NC
kDa
kDa
130
100
ACSL4
70
100
AGO1
70
55
PRKAA2
55
β-Actin
β-Actin
40
40
D
Si-NC
Si-AGO2-1
Si-AGO2-2
-
-
-
+
+
+
miR-214
Si-AGO2-2
Si-AGO2-1
kDa
Si-NC
kDa
100
ACSL4
130
70
AGO2
100
PRKAA2
70
70
55
β-Actin
55
40
β-Actin
40
